# Integrated metabolomic and transcriptomic profiling uncovers a citrus terpene synthase with *in vivo*–*in vitro* catalytic divergence and psyllid-attracting ecological function

**DOI:** 10.3389/fpls.2026.1911599

**Published:** 2026-09-29

**Authors:** Qihuan Deng, Qingqing Zi, Chao Yang, Haoning He, Yaohai Zhang, Qiying Wang, Xiaochun Wang, Yuan Wang, Hongfeng Tian, Huayu Yang, Xinyuan Wu, Yaobin Lv, Xuefeng Wang, Changyong Zhou, Jun He

**Affiliations:** 1 Integrative Science Center of Germplasm Creation in Western China (Chongqing) Science City, Citrus Research Institute, Southwest University, Chongqing, China/National Citrus Engineering Research Center, Citrus Research Institute, Southwest University, Chongqing, China; 2 Xianghu Laboratory, Hangzhou, China

**Keywords:** citrus, *Diaphorina citri*, hairy roots, huanglongbing (HLB), methyl jasmonate (MeJA), terpenoid biosynthesis

## Abstract

**Introduction:**

Citrus plants produce a rich diversity of volatile organic compounds (VOCs) that play important roles in fruit aroma, essential oils, and defense against biological stresses in aboveground tissues, yet volatile profiles in citrus roots remain poorly understood.

**Methods:**

Here, we employed an integrated metabolomic and transcriptomic approach to investigate VOCs and associated genes in Carrizo citrange (*Citrus sinensis* × *Poncirus trifoliata*) roots, by comparing control roots (CK), *Rhizobium rhizogenes*-induced hairy roots (HR) and those further elicited by methyl jasmonate (HR+MeJA).

**Results:**

We identified 642 VOCs in the root samples, with terpenoids representing the most dominant chemical class. Comparative analysis of differential metabolites and genes between HR, HR+MeJA, and controls revealed terpenoids and candidate genes that were responsive to *R. rhizogenes* induction and MeJA elicitation. Among them, a putative terpene synthase gene, *Cs2g07240*, was found to be highly transcribed in several aboveground tissues and markedly upregulated upon Huanglongbing (HLB) infection. *In vitro* heterologous expression verified that this gene converted GPP to linalool and FPP to trans-nerolidol. However, overexpressing this gene in Carrizo citrange hairy roots promoted linalool and several non-target terpenes but not trans-nerolidol. Furthermore, stable overexpression of *Cs2g07240* in sweet orange (*C. sinensis*) increased linalool accumulation in leaves, whereas trans-nerolidol remained undetectable. Leaves of the overexpression lines were also more attractive to adults of *Diaphorina citri*, the insect vector of HLB.

**Discussion:**

Collectively, this study unveils the terpenoid volatile repertoire in citrus roots and its modulation by *R. rhizogenes* and MeJA, while also highlighting the catalytic divergence of this synthase between *in vitro* and *in planta* contexts, as well as the tissue-specific complexity of terpenoid biosynthesis and ecological function.

## 1 Introduction

Citrus is among the most economically significant horticultural crops globally, valued for its nutritional content, commercial versatility, and cultural importance. A defining feature of *Citrus* species is their extensive diversity of volatile organic compounds (VOCs), which play multifaceted roles in determining fruit quality, aroma profiles, and essential oil composition (Dosoky and Setzer, 2018). These VOCs directly influence consumer preference by contributing to characteristic flavors and fragrances, while also serving vital ecological functions, such as attracting pollinators and enhancing reproductive success (Mohammed et al., 2020; Alsabte et al., 2022). A Furthermore, citrus-derived VOCs, particularly terpenoids, function as key chemical mediators in plant-environment interactions, providing natural defense mechanisms against pathogens and herbivores through their antimicrobial and repellent properties (Rodríguez et al., 2014). Industrially, citrus VOCs are extracted for applications in food additives, cosmetics, and phytopharmaceuticals, underscoring their agricultural and commercial importance (Pandey et al., 2023; Grover et al., 2025).

Terpenoids are the largest class of compounds in plant VOCs. Composed of isoprenoid units, volatile terpenoids exhibit highly diverse structures, ranging from hemiterpenoids (C_5_), monoterpenoids (C_10_) to sesquiterpenoids (C_15_) (Gershenzon and Dudareva, 2007). These compounds underpin critical biological functions, including the attraction of pollinators, the deterrence of herbivores (Reisenman et al., 2013; He et al., 2021), and the recruitment of natural enemies of pests (Qin et al., 2024; Wei et al., 2024). Furthermore, volatile terpenoids are essential for the inhibition of phytopathogens (Gajera et al., 2016; Qiao et al., 2020; Javed et al., 2021; Mirowski et al., 2023) and act as pivotal mediators in systemic defense signaling, often regulated by phytohormones in response to environmental stimuli (Maleki et al., 2024). Typically, terpenoids are biosynthesized via the mevalonate (MVA) and 2-*C*-methyl-D-erythritol 4-phosphate (MEP) pathways, which generate the universal precursors geranyl diphosphate (GPP) and farnesyl diphosphate (FPP). These precursors are subsequently converted by terpene synthases (TPSs) into a vast array of scaffolds with immense structural complexity (Upadhyay et al., 2025).

Terpenoids in *Citrus* species are extensively distributed across various organs, including leaves, and epidermal tissues, where they function as critical mediators of disease resistance, pest deterrence, and flavor regulation. For instance, the monoterpene (+)-limonene, abundant in fruits and leaves, has been shown to enhance resistance to citrus canker (*Xanthomonas citri*) by inducing callose deposition (Shimada et al., 2014; Saini et al., 2022). The sesquiterpene α-copaene repels Asian citrus psyllid (ACP, *Diaphorina citri*) at ~100-fold lower doses than β-caryophyllene. A 1:100:10 blend of α-humulene, α-copaene, and β-elemene further enhances repellency, significantly reducing HLB transmission risk (Magnani et al., 2025). Flavor profiles of citrus fruits are largely shaped by terpenoids such as γ-terpinene and valencene, which contribute to their characteristic aroma (Huang et al., 2017). Notably, the sesquiterpene β-caryophyllene in citrus volatiles attracts adult psyllids, inadvertently facilitating HLB spread, highlighting the dual ecological roles of terpenoids in both defense and pathogen dissemination (Alquézar et al., 2021). At the molecular level, several *TPS* genes have been isolated and characterized, including *TPS*1(*Ocimum kilimandscharicum*), responsible for limonene synthesis (Anand et al., 2019), and *CsTPS*1, involved in valencene production as a precursor to nootkatone (Sharon-Asa et al., 2003). Previous studies identified 49 TPS genes in Citrus species, revealing their roles in terpenoid biosynthesis and adaptation (Dornelas and Mazzafera, 2007); in addition, genomic analysis of *C. sinensis* unveiled 95 TPS loci (55 functional) spanning all angiosperm subfamilies and validated defense-linked enzymes like β-farnesene synthase (Alquézar et al., 2017).

While VOCs in aboveground citrus tissues are well-characterized, their roles in citrus roots remain largely unexplored. Root-emitted VOCs play critical roles in mediating belowground ecological interactions, including herbivore deterrence, microbial communication, and soil-borne defense coordination, with terpene synthases (TPSs) serving as key biosynthetic regulators of these metabolites (Gfeller et al., 2019; Jones and Killiny, 2021). Crucially, recent evidence suggests that volatile-biosynthetic genes often exhibit distinct, tissue-specific expression patterns—frequently showing higher baseline abundance in foliage or pericarps compared to belowground organs. In aerial tissues, these localized volatile profiles dictate crucial multi-trophic relationships; for instance, Huanglongbing (HLB) infection significantly alters leaf VOC emissions, which directly modulates the olfactory attraction of the insect vector, the citrus psyllid (*Diaphorina citri*) (Pérez-Hedo et al., 2024). However, whether the core biosynthetic pathways and defense-responsive volatile genes discovered in citrus roots play functionally conserved yet localized ecological roles when expressed in different plant compartments—for example, reconfiguring leaf volatilomes to against aerial pests—remains uncharacterized. To resolve this mystery, it is essential to systematically profile the citrus root volatilome for primary target gene discovery, and subsequently evaluate the independent, localized contributions of these candidate genes within aboveground pest interactions.

*R. rhizogenes*-mediated genetic transformation and subsequent induction of hairy root provide advantageous platforms for investigating plant secondary metabolism (Sevón and Oksman-Caldentey, 2002; Georgiev et al., 2010; Häkkinen and Oksman-Caldentey, 2018). Importantly, for perennial woody species such as citrus, which are characterized by long juvenile phases and low transformation efficiency, the HR system serves as an indispensable high-throughput platform for rapid functional genomics. While traditional *Agrobacterium*-mediated citrus transformation can take years to produce mature transgenic lines, HR induction allows for the rapid functional characterization of candidate genes—such as terpene synthases (TPSs)—within months. By mimicking natural root stress responses through the activation of JA/auxin crosstalk and ROS signaling (Robert-Seilaniantz et al., 2011; Bittner et al., 2022; Zheng et al., 2025), the HR system provides a biologically relevant context for deciphering complex metabolic networks. Consequently, utilizing HR as a ‘fast-track’ screening tool followed by targeted whole-plant validation offers a powerful strategy to link candidate genes initially identified from root transcriptomes with systemic ecological phenotypes, such as plant-insect interactions.

This study aimed to characterize root VOC profiles and associated transcriptional changes in Carrizo citrange (*Citrus sinensis* × *Poncirus trifoliata*) following hairy-root induction and MeJA treatment. We further aimed to characterize the biochemical function of *Cs2g07240* and assess how its overexpression affects volatile accumulation in hairy roots and stable transgenic sweet orange leaves. The olfactory responses of *D. citri* adults to the transgenic leaves were also evaluated. In total, 642 putative metabolites were identified from those roots, with terpenoids representing the dominant chemical class. Multi-omics integrative analysis demonstrated that both *R. rhizogenes* transformation and MeJA treatment substantially remodeled terpenoid accumulation and the transcription of their biosynthetic pathway genes. One target gene, *Cs2g07240*, was highly expressed in multiple aboveground tissue and markedly induced upon Huanglongbing (HLB) infection. Functional characterization via heterologous *in vitro* expression verified that its encoded enzyme catalyzes the synthesis of linalool and nerolidol. Nevertheless, in hairy roots overexpressing *Cs2g07240*, only the production of linalool and several other terpenoid volatiles was significantly elevated but not nerolidol. In addition, leaves of stable *Cs2g07240*-overexpressing sweet orange plants via *Agrobacterium tumefaciens*-mediated transformation exhibited higher linalool accumulation and stronger attractiveness to *Diaphorina citri*, the sole insect vector transmitting HLB pathogens.

## 2 Materials and methods

### 2.1 Plant growth and *R. rhizogenes* induced hairy roots generation

Epicotyl segments (1-cm explants) were collected from ~2-year-old Carrizo citrange (*Citrus sinensis* × *Poncirus trifoliata*) plants grown in Chongqing, China. Hairy roots (HRs) were induced using *R. rhizogenes* strain MSU440 harboring the binary vector pNm*GFP*er, following the protocol described by Irigoyen et al. (2020) with slight modifications (Irigoyen et al., 2020). Briefly, *R. rhizogenes* cells were cultured in tryptone yeast extract medium (TY) liquid medium with appropriate antibiotics at 28°C to an OD_600_ of 0.6–0.8. Bacterial cells were harvested by centrifugation, resuspended in MS liquid medium (OD_600_ = 0.6~0.8), and used for vacuum infiltration of the plant explants for 30 min. Mock-treated explants served as controls. Following inoculation, all explants were vertically embedded in sterile vermiculite and maintained in a growth chamber at 26 °C under an 80% relative humidity and a 16-h photoperiod. After a 20-day cultivation period, the successful induction of transgenic hairy roots was confirmed through a dual verification approach, involving simultaneous *GFP* fluorescence screening and polymerase chain reaction (PCR) analysis. The PCR analysis primer sequences are detailed in **Supplementary Table S1**.

### 2.2 Treatment of MeJA

The day prior to elicitation, the roots not infected by *R. rhizogenes* (CK) and HR (divided into two groups: HR control and HR+MeJA) were placed in three hydroponic incubators containing modified Hoagland nutrient solution for 24 hours. At 8 am on the following day, one hairy roots group designated for treatment was exposed to MeJA (Macklin, 98%, China, CAS: 39924-52-2) at a final concentration of 200 μM in the liquid medium. At 12:00 pm of the day, samples from the hydroponic incubators were collected into 50 mL centrifuge tubes and immediately flash-frozen in liquid nitrogen and subsequently stored at -80 °C until further analysis. Each of the three experimental groups (CK, HR control and HR+MeJA) included three biological replicates.

### 2.3 Sample preparation and GC-MS analysis of VOCs

For the CK, HR, and HR+MeJA metabolomic comparison, three biological replicates were analyzed per group. Each biological sample was analyzed once by GC-MS, without additional technical replicate analyses. Harvested plant materials were ground to a fine powder in liquid nitrogen. A 500 mg aliquot of the powder was immediately transferred to a 20 mL headspace vial containing a saturated NaCl solution to inhibit endogenous enzymatic activity. For relative quantification, 20 µL of internal standard (IS-04, 10 µg/mL) was added to each sample prior to sealing. Headspace solid-phase microextraction (HS-SPME) was executed at 60°C for 15 min using a 120 µm DVB/CWR/PDMS fiber (Agilent) following a 5-min pre-heating step. Volatile compounds were subsequently desorbed in the injector port of an Agilent 8890 GC system at 250°C for 5 min in splitless mode.

GC-MS analysis was conducted on an Agilent 8890 GC coupled with a 7000D mass spectrometer equipped with a DB-5MS capillary column (30 m × 0.25 mm × 0.25 μm). Helium with a purity of at least 99.999% was used as the carrier gas at a flow rate of 1.2 mL/min. The oven temperature program was configured as follows: 40°C (3.5 min), ramped at 10 °C/min to 100 °C, then at 7°C/min to 180 °C, and finally at 25°C/min to 280°C (held for 5 min). Mass spectra were recorded in electron impact ionization mode at 70 eV using selected ion monitoring (SIM) mode. The ion-source, quadrupole, and transfer-line temperatures were set to 230, 150, and 280 °C, respectively.

The internal-standard-normalized relative abundance of each putatively identified volatile compound (X_i_, expressed as µg/g fresh weight) was calculated using the internal standard according to the following equation:


$$X_{i}=\frac{V_{s}\times C_{s}}{M/V}\times \frac{I_{i}}{I_{s}}\times 10^{-3}$$

where *Vs* (20 µL) and *Cs* (10 µg/mL) represent the volume and concentration of the internal standard (IS-04), *M* (or *V*) represents the weight (or volume) of the sample, and *I_i_* and *I_s_* are the peak areas of the target compound and the internal standard, respectively. As authentic standards were not used for all detected compounds, this internal-standard-based approach provides semiquantitative estimates for comparing the abundance of individual VOCs among the CK, HR, and HR+MeJA groups.

### 2.4 Transcriptome sequencing and DEG analysis

Total RNA was extracted from frozen root samples of Carrizo citrange in the CK, HR, and HR+MeJA groups, with three biological replicates per group. Sequencing libraries were prepared using the NEBNext^®^ Ultra™ RNA Library Prep Kit for Illumina^®^ (NEB, USA) for each sample, following the manufacturer’s instructions. For data analysis, raw paired-end reads were subjected to quality control. Clean reads were then mapped to the reference genome and its corresponding annotation files, which were obtained from a designated public repository. HISAT2 was employed to construct the genome index, and subsequently, to align the quality-filtered reads to the indexed reference genome. Gene-level read counts were quantified using featureCounts. Fragments per kilobase of transcript per million mapped reads (FPKM) values were then calculated for each gene, normalizing for gene length and sequencing depth.

### 2.5 Principal component analysis, gene ontology and genes and genomes enrichment analysis of differential metabolites and differentially expressed genes

Unsupervised principal component analysis (PCA) and orthogonal partial least squares-discriminant analysis (OPLS-DA) were performed in R. Differentially accumulated metabolites (DAMs) were screened based on variable importance in projection (VIP) scores > 1 and (|Log_2_FC|) ≥1.0 derived from the OPLS-DA model (200 permutations). Differentially expressed genes (DEGs) were identified using the DESeq2 package with unnormalized gene-level RNA-seq read counts as input, using thresholds of Benjamini–Hochberg-adjusted *p* < 0.05 and |log2FC| ≥ 1.0. For both DEGs and DAMs, upregulation and downregulation refer to increases and decreases in HR relative to CK and in HR+MeJA relative to HR, respectively. Both DEGs and DAMs were annotated against the KEGG and GO databases, and metabolic pathways or functional categories were subjected to over-representation enrichment analysis using the hypergeometric test (*p* < 0.05).

### 2.6 Combined analysis of RNA-seq and GC-MS

Pearson correlation analysis was performed between log_2_-transformed gene-expression levels of DEGs and metabolite-abundance values of DAMs using the cor package in R. Co-enriched metabolic pathways were identified through comparative KEGG enrichment analysis. For direction-specific analysis, DEGs and DAMs were classified as upregulated or downregulated according to their fold changes relative to the designated reference group in each pairwise comparison. KEGG enrichment analyses were then performed separately for the upregulated and downregulated subsets of DEGs and DAMs in each comparison using the hypergeometric test. The resulting p-values were adjusted for multiple testing using the Benjamini–Hochberg method. Highly correlated gene-metabolite pairs (|Pearson *r*| > 0.8, *p* < 0.05) were filtered and utilized to construct a bipartite correlation network in Cytoscape, where nodes represent genes or metabolites and edges reflect correlation coefficients. Functionally relevant DEG-DAM associations were subsequently mapped to terpenoid biosynthesis and JA signaling pathways using KEGG Mapper to elucidate mechanistic linkages between transcriptional regulation and metabolic reprogramming.

### 2.7 Reverse-transcription quantitative PCR analysis

Total RNA was re-extracted using RNAiso Plus (TaKaRa) from the same biological root samples of Carrizo citrange in the CK, HR, and HR+MeJA groups that had been used for RNA-seq and returned by the sequencing service provider. Three biological replicates were analyzed for each group. Reverse transcription using All-In-One 5× RT Master Mix (ABM) was performed following the manufacturer’s instructions. To validate the RNA-seq results, qRT-PCR analysis was performed on selected genes using equal concentrations of synthesized cDNA. qPCR was performed using a q225 real-time PCR machine (Novogene) and a SYBR Prime qPCR set (Bioground). The reactions were carried out with SYBR Green PCR Master Mix (ABM), with primer sequences detailed in **Supplementary Table S1**. Each biological replicate was analyzed using three technical PCR replicates. The actin gene was used as an internal reference gene, and relative expression was quantified using the 2^−ΔΔCt^ method (Wang et al., 2024). Statistical analysis and plotting were performed using GraphPad Prism v. 10.1.2 software.

### 2.8 Boosting terpenoid biosynthesis through overexpressing terpenoid synthase genes in HR of transgenic *C. citrange*

The full-length coding sequence (CDS) of *Cs2g07240* was amplified from citrus cDNA and cloned into the *BamHI* and *KpnI* sites of the pNm*GFP*er expression vector via homologous recombination. Carrizo citrange explants were transformed with *R. rhizogenes* strain MSU440 harboring either pNmGFPer-*Cs2g07240* or the corresponding empty vector, following the procedure described in Section 2.1. Hairy roots transformed with the empty vector served as the control (EV-HR). GFP-positive hairy roots were selected after six weeks, and transgene integration was confirmed by genomic PCR. Before GC-MS analysis, both the empty-vector control and *Cs2g07240*-overexpressing hairy roots were transferred to modified Hoagland nutrient solution for 24 h and subsequently treated with 200 μM MeJA for 4 h under identical conditions. Samples were collected at 12:00, immediately frozen in liquid nitrogen, and stored at -80°C. Three independent biological replicates were analyzed for each group. Terpenoid volatiles were analyzed by GC-MS as described in Section 2.3. Data are presented as the mean ± SD, and differences between the two groups were evaluated using a two-tailed unpaired Student’s t-test in GraphPad Prism. Primer sequences are provided in **Supplementary Table S1**.

### 2.9 *In vitro* enzymatic characterization of *Cs2g07240*.

The full-length CDS of *Cs2g07240* was cloned into the pET28a vector and heterologously expressed in *E. coli* Rosetta 2(DE3) cells (He et al., 2019). *In vitro* enzymatic assays were conducted by incubating 40 μL of purified protein with substrates (GPP or FPP) and *MgCl*_2_ at 30°C for 2 h. An equivalent protein preparation from cells carrying the empty pET28a vector was assayed under identical conditions as the negative control. Reaction products were extracted with *n*-hexane and analyzed via GC-MS (HP-5MS column) using EI mode (70 eV). Authentic linalool and trans-nerolidol standards were analyzed under identical GC-MS conditions. Product retention times and mass spectra were compared with those of the authentic standards. Terpenoid identification was performed by comparing mass spectra against NIST 08 and Flavour 2.0 libraries (match factor ≥ 90%).

### 2.10 Generation of stable transgenic citrus overexpressing *Cs2g07240*

The plant expression vector pCAMBIA-*Cs2g07240* driven by the CaMV 35S promoter was constructed and introduced into *Agrobacterium tumefaciens* strain EHA105. Stable transgenic sweet orange plants were generated using shoot segments following the method described by Peña et al (Pena et al., 1995). Plants transformed with the corresponding empty pCAMBIA vector were used as the empty-vector control (EV). Positive lines (OE_1, OE_2, OE_3) were confirmed by GUS staining, genomic PCR, and RT-qPCR. For RT–qPCR verification of *Cs2g07240* overexpression, leaf samples were collected from the three independent transgenic lines (OE_1, OE_2, and OE_3) and the corresponding EV plants. For each transgenic line and the EV control, three leaf samples were analyzed by RT-qPCR. Each sample was assayed in three technical replicates. RT-qPCR was performed as described in Section 2.7, with three technical replicates per biological sample. Expression was normalized to CitActin and calculated relative to the EV control. Leaf volatiles from transgenic and EV plants were extracted with *n*-hexane and quantified via GC-MS (section 2.9) using peak area normalization. Primer sequences are listed in **Supplementary Table S1**.

### 2.11 HLB-associated expression analysis of *Cs2g07240* and behavioral assays of *Diaphorina citri*

To assess HLB-associated expression of *Cs2g07240*, fully expanded leaves were collected from healthy and HLB-infected ‘Wanjincheng’ sweet orange (*Citrus sinensis*) plants. Infection status was confirmed by PCR. Three biological replicates were analyzed per group. RT-qPCR was performed as described in Section 2.7, using *CitActin* as the reference gene and the healthy group as the calibrator. Olfactory preferences of *D. citri* adults were evaluated using a glass Y-tube olfactometer with symmetrical airflow adjusted to 400 mL/min per arm. In the synthetic compound assay, 20 μL of synthetic linalool solution (1 μL/mL in *n*-hexane) or 20 μL of *n*-hexane was applied to filter paper in the corresponding odor chamber, testing 20 responsive psyllids per replicate across three independent runs (*n* = 60 choices in total). In the plant tissue assay, 5 fresh leaves from each *Cs2g07240*-overexpressing line (OE_1, OE_2, and OE_3) were separately paired with five leaves from the corresponding EV plants. Each OE-line comparison included 48 responsive psyllids, yielding 144 valid choices across the three lines. A choice was recorded when an insect crawled beyond half the length of either lateral arm and remained there for at least 30 s within the 10-min observation period. To minimize directional and residual odor biases, the Y-tube was rotated by 180° after every eight insects and replaced with a clean tube after every 48 insects. The pooled choice counts were compared with an expected 1:1 distribution using a chi-square (χ²) test.

## 3 Results

### 3.1 Generation of *R. rhizogenes* induced and MeJA stimulated citrange hairy roots

To generate induced HR and those further stimulated by MeJA (HR+MeJA), an overexpression vector pNm*GFP*er carrying a *mGFP* (**Figure 1A**) was introduced into *R. rhizogenes* and then used for transformation of citrange stem explants. 30 days later, we found that the transformed plants had grown significantly more roots than the controls (CK). These roots were checked under UV light which excited *GFP*-derived green fluorescence (**Figure 1B**). Most of the induced HR and HR+MeJA exhibited green fluorescence while CK roots did not, indicating successful transformation and introduction of the *mGFP*. These positive roots were further confirmed by PCR amplification of the *rolB* and *rolC* genes from *R. rhizogenes* and the *mGFP* transgene. No amplification products were observed for CK samples, whereas all three target genes were detected in both HR and HR+MeJA samples (**Figure 1C**). These results indicate successful generation of induced transgenic HR expressing exogenous *GFP* gene.

**Figure 1:**
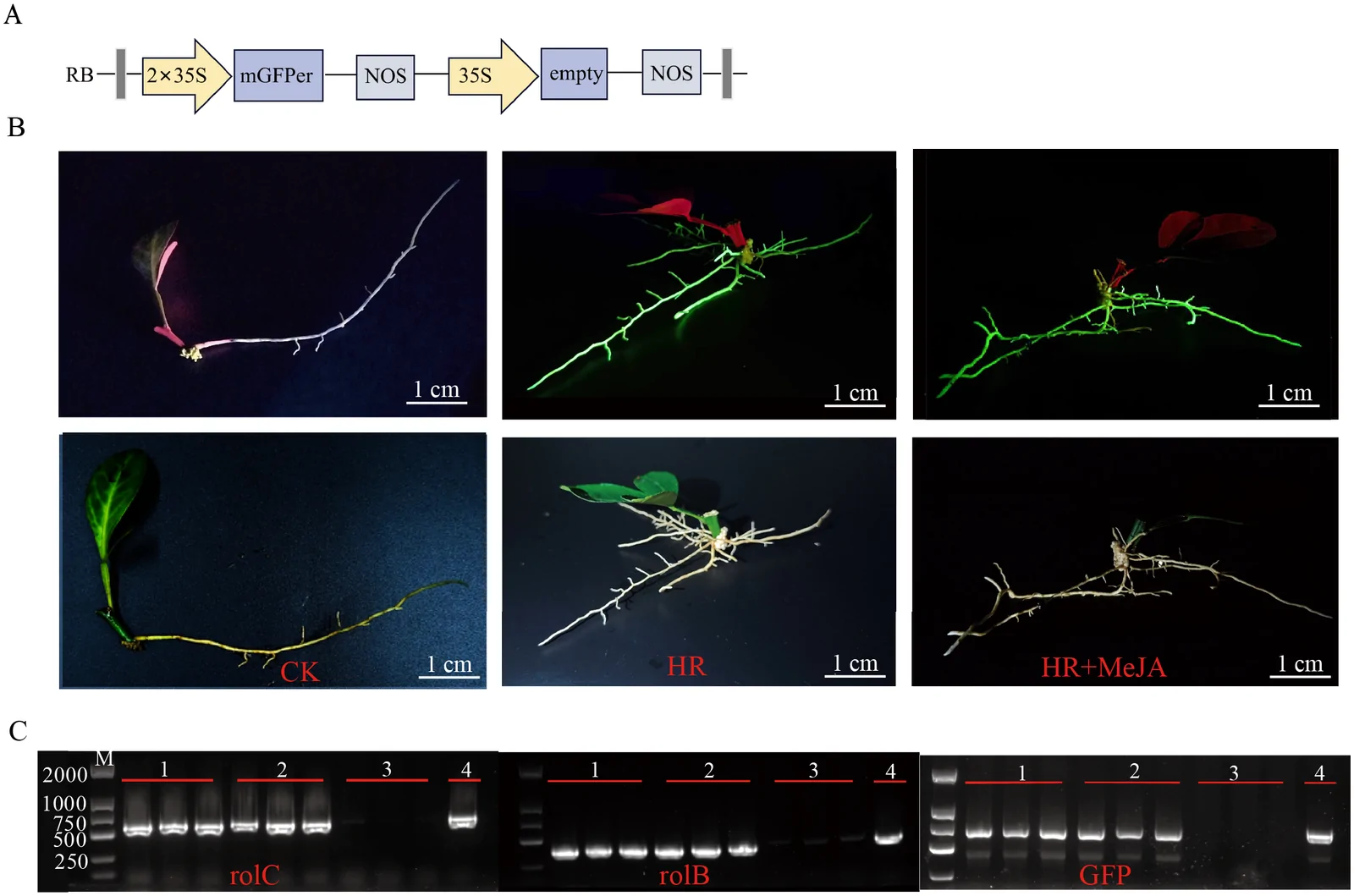
Generation and molecular characterization of citrange hairy roots (HR) induced by *Rhizobium rhizogenes* (*R. rhizogenes*) and that stimulated by methyl jasmonate (MeJA) treatment (HR+MeJA). **(A)** Schematic diagram of the T-DNA region in a binary vector pNm*GFP*er which was used for HR induction; *mGFP* driven by CaMV35S promoter was used to indicate successful transformation. NOS, nopaline synthase terminator. **(B)** Detection of GFP fluorescence in CK, HR and HR+MeJA under UV light. Upper panel: UV light, lower panel: bright light, Scale bars, 1 cm. **(C)** PCR verification for the presence of *rolC*, *rolB* and *GFP* (473 bp, 450 bp, 724 bp bands) in CK, HR and HR+MeJA. M: D2000 DNA marker; 1: HR, 2: HR+MeJA, 3: CK, 4: plasmid DNA.

### 3.2 Metabolomic analysis of HR and HR+MeJA

GC-MS analysis captured a total of 642 volatile organic compounds (VOCs), across the CK, HR, and HR+MeJA groups, spanning 16 chemical classes (**Figure 2A**; **Supplementary Table S3**). Among these compounds, there were 203 terpenoids, taking the biggest group and 31.62% of the total detected compounds (**Figure 2A**). Moreover, in abundance these terpenoids accounted for 41.08%, 41.96% and 42.87% of the detected VOCs in CK, HR, and HR+MeJA, respectively (**Supplementary Figures S1A–C**). Notably, aldehydes ranked as the second most prevalent category, maintaining a substantial and consistent presence of approximately 20% across all groups, whereas minor constituents such as halogenated hydrocarbons remained at trace levels below 0.01% (**Supplementary Figure S1**).

**Figure 2:**
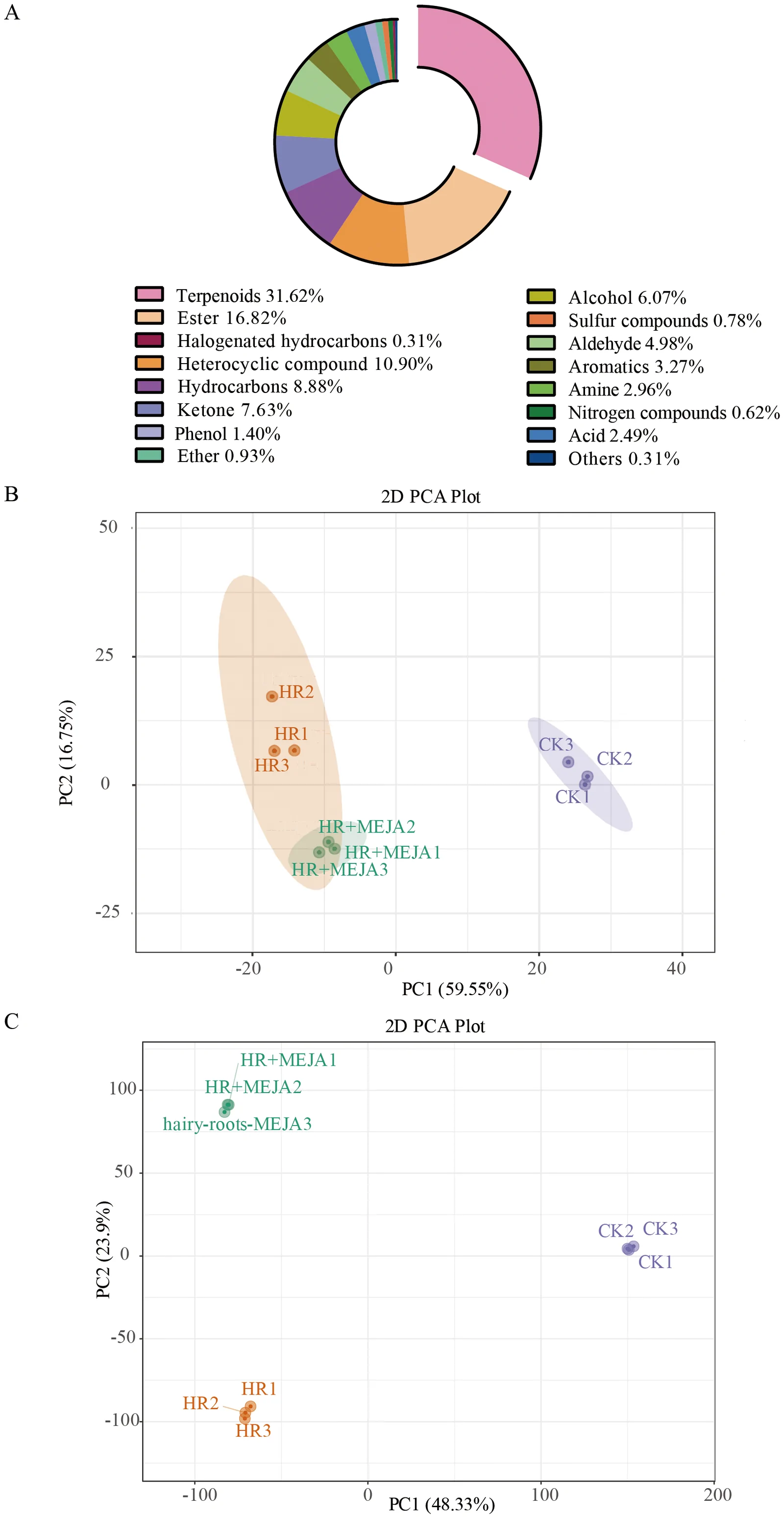
Overview of volatile organic compound (VOC) and transcriptome profiles in control roots (CK), *R. rhizogenes-*induced hairy roots (HR), and MeJA-treated hairy roots (HR+MeJA). **(A)** Chemical classification of the non-redundant VOCs detected across all three groups. The percentage of each chemical class was calculated by dividing the number of distinct VOCs assigned to that class by the total number of classified distinct VOCs and multiplying by 100. **(B)** Principal component analysis (PCA) of VOC abundance profiles. **(C)** PCA of transcriptome profiles. Each point in **(B)** and **(C)** represents one biological replicate.

PCA of the three groups of samples based on the detected compounds demonstrated clear separated clustering of different treatments: the first principal component (PC1, 59.55% variance) effectively segregated *R. rhizogenes*-induced hairy roots from non-induced controls, while the second principal component (PC2, 16.75% variance) discriminated samples by MeJA treatment (**Figure 2B**). Notably, transcriptomic PCA demonstrated analogous clustering patterns, with PC1 (48.33% variance) achieving precise differentiation of *R. rhizogenes*-induced hairy root induction status, and PC2 (23.90% variance) successfully distinguishing molecular responses to MeJA elicitation (**Figure 2C**).

Differentially accumulated metabolites (DAMs) were identified based on OPLS-DA-derived VIP values > 1 and an absolute log2 fold-change threshold ≥ 1.0. Substantial changes in the metabolomic profiles were observed in HR compared with CK, with 53 DAMs upregulated and 70 downregulated (**Figure 3A**). The comparison between HR+MeJA and HR identified 21 upregulated and 0 downregulated DAMs (**Figure 3B**).

**Figure 3:**
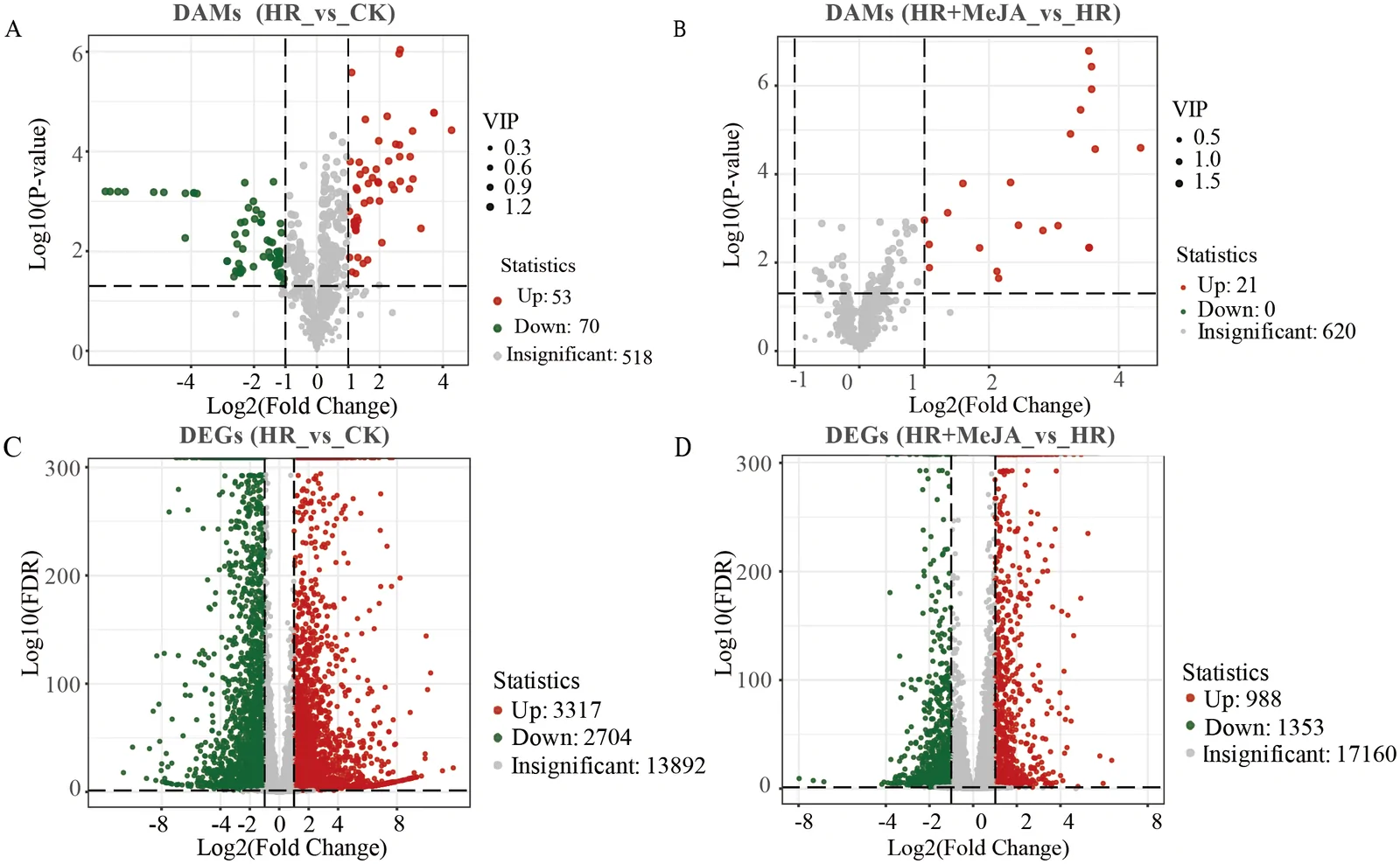
Differentially accumulated metabolites (DAMs) and differentially expressed genes (DEGs) detected between CK, HR and HR+MeJA. **(A, B)** DAMs in comparison of HR _vs_CK **(A)** and HR +MeJA_vs_HR **(B)**. **(C, D)** DEGs in comparison of HR_vs_CK **(C)** and HR+MeJA_vs_ HR **(D)**. Variable importance in projection (VIP) scores measure the contribution of each metabolite to group separation in the OPLS-DA model. Metabolites with |log2FC|≥1 and VIP > 1 are considered significantly differential.

### 3.3 Transcriptomic analysis of HR and HR+MeJA

To reveal the transcriptome reprogramming associated with the VOCs alteration during the *R. rhizogenes* induction and MeJA stimulation, we profiled the gene expression in CK, HR and HR+MeJA samples using RNA-seq. Following rigorous data quality control, we obtained a total of 62.42 GB of high-quality clean sequencing data. Each sample yielded at least 6 GB of clean data, with Q30 base percentages exceeding 93% and GC content ranging between 44.435% and 44.46%. These metrics considered to be high reliability of the data for subsequent analyses. The complete dataset is presented in **Supplementary Table S2**.

Transcriptomic analysis revealed distinct gene expression patterns across experimental groups. Pairwise comparisons showed that HR vs CK exhibited 3,317 upregulated and 2,704 downregulated genes, while HR+MeJA vs HR displayed 1,353 upregulated and 988 downregulated genes. Specifically, 6,021 DEGs were identified between CK and HR using HR as the reference group (3,317 upregulated, 2,704 downregulated). Similarly, HR+MeJA vs HR comparisons detected 2,341 DEGs (1,353 upregulated, 988 downregulated) under the same reference condition (**Figures 3C, D**).

### 3.4 Integrated -omics analysis revealed key pathways regulated in HR and HR+MeJA

Integrated analysis of DEGs and DAMs revealed distinct KEGG pathway enrichment patterns among CK, HR, and HR+MeJA (**Figure 4**). The HR vs. CK comparison showed significant enrichment in 10 metabolic pathways (*p* < 0.05), predominantly associated with “Metabolic pathways” and “Biosynthesis of secondary metabolites”. Notably, specialized pathways including folate, benzoxazinoid, and isoquinoline alkaloid biosynthesis were markedly upregulated, alongside specific activation of the sesquiterpenoid/triterpenoid and diterpenoid biosynthetic pathways (**Figure 4A**). In contrast, MeJA elicitation (HR+MeJA vs. HR) was associated with enrichment of the terpenoid backbone biosynthesis pathway, with sesquiterpenoid/triterpenoid biosynthesis represented in both comparisons (**Figure 4B**). Separate integrated KEGG enrichment analyses of upregulated and downregulated features provided additional insight into these overall patterns (**Supplementary Figure S2**). The integrated analysis associated benzoxazinoid and diterpenoid biosynthesis with upregulated features and folate and isoquinoline alkaloid biosynthesis with downregulated features in HR vs. CK, whereas terpenoid backbone biosynthesis was associated with upregulated features in HR+MeJA vs. HR (**Supplementary Figure S2**).

**Figure 4:**
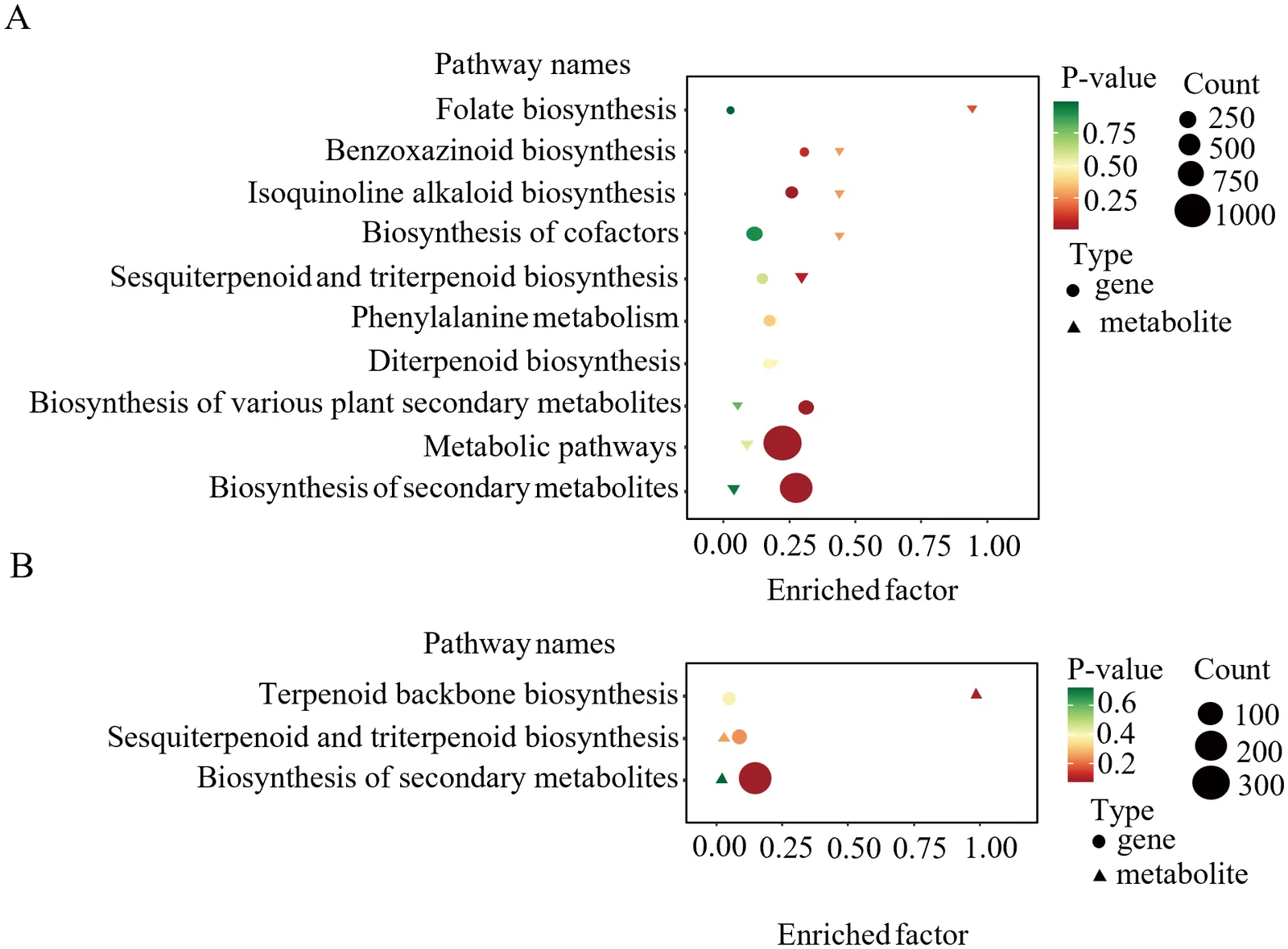
Top KEGG Pathways differentially affected in HR and HR+MeJA revealed by integrated analysis of differentially accumulated metabolites (DAMs) and differentially expressed genes (DEGs) between **(A)** HR_vs_CK and **(B)** HR+ MeJA_vs_HR. The graphical representation employs bubbles to delineate different-omics data. Circles represent the number of genes, while triangles represent the number of metabolites, and bubble size is proportional to the number of statistically significant features (DEGs or DAMs) identified within each omics dataset.

This metabolic rewiring was mechanistically supported by concurrent transcriptional reprogramming (**Supplementary Figures S3, S4**). GO enrichment analysis revealed that HR roots exhibited enhanced activation of responses to oxidative stress, auxin signaling, and isoprenoid/terpenoid biosynthetic processes compared to CK controls (**Supplementary Figure S3A**). MeJA treatment further potentiated these defense strategies, heavily prioritizing hormone homeostasis and signal transduction modulation (**Supplementary Figure S3B**). Furthermore, KEGG mapping confirmed that while phenylpropanoid and flavonoid biosynthesis dominated the baseline HR profile (**Supplementary Figure S4A**). MeJA elicitation uniquely shifted metabolic priorities toward ubiquinone and terpenoid-quinone biosynthesis (**Supplementary Figure S4B**), defining the regulatory transition from generic root transformation to targeted jasmonate-induced defense.

### 3.5 Terpenoid biosynthesis and related genes were regulated in HR and HR+MeJA

Hierarchical cluster analysis of 71 differential terpenoid volatiles across CK, HR, and HR+MeJA samples classified them into four groups (**Figure 5A**; **Supplementary Table S7**). Cluster 1 (21 compounds, from (*Z*)-farnesene to (-)-γ-elemene) showed high abundance in CK but decreased both in HR and HR+MeJA. These cluster includes both monoterpenes [e.g., 3-carene and (+)-α-pinene] and sesquiterpenes (e.g., α-cadinene, *trans*-caryophyllene, γ-selinene). Some them including β-(*Z*)-farnesene, β-germacrene and (-)-α-guriunene were rescued a little bit in HR+MeJA samples. Cluster 2 [12 compounds from selina-3,7(1 l) to α-cedrene] displayed high abundance in CK, HR but low in HR+MeJA. This cluster includes sesquiterpenes ϵ-muuroloene, β-longipinene, α-zingiberene, (*E*, *E*)-α-farnesene and α-copaene. Conversely, Cluster 3 (34 compounds, the largest group containing β-pinene and (+)-limonene) was strongly induced in both HR and HR+MeJA groups. Cluster 4 (4 compounds, including alloocimene and β-sabinene) remained at low levels in both CK and HR but was uniquely stimulated by MeJA elicitation.

**Figure 5:**
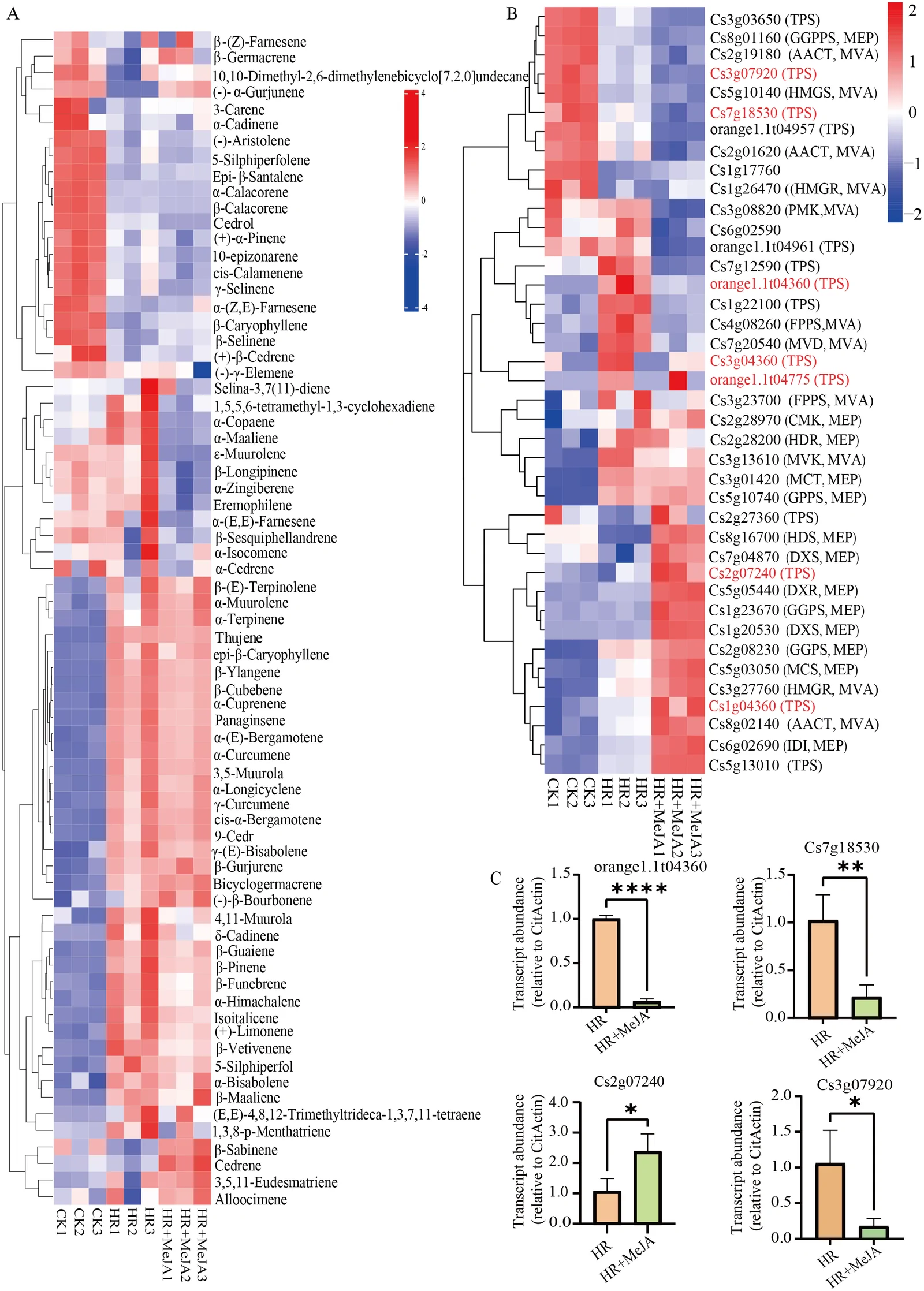
The Regulation of terpenoid biosynthesis by methyl jasmonate (MeJA) treatment in citrange hair roots. **(A)** Clustering analysis of terpenoids accumulated in control (CK), hairy roots (HR) and HR+MeJA. **(B)** Heatmap analysis of transcription level of terpenoid biosynthesis related genes. **(C)** Validation of the transcript levels of key MeJA-responsive terpene synthase genes using RT-qPCR. Values are the log2-fold changes compared with the average expression in noninduced roots. Light red indicates upregulation and dark red indicates strong upregulation of gene expression. Blue indicates downregulation of gene expression levels and a strong downregulation is indicated in dark blue. Transcript per million mapped reads (FPKM) values are presented in Supporting Information **Supplementary Table S7**. The differences between samples of the different stimulated groups were analyzed using Student’s t-test (for two-group comparisons). *, **, and *** indicate significant difference at *p* < 0.05, *p* < 0.01, and *p* < 0.0001, respectively.

Transcriptome analysis identified 40 putative genes associated with terpenoid biosynthesis, including genes involved in MVA- and MEP-dependent precursor biosynthesis (Vranová et al., 2013), TPS genes involved in terpene formation (Chen et al., 2011). Their expression profiles were examined by hierarchical clustering (**Figure 5B**; **Supplementary Figure S5**; **Supplementary Table S5**), revealing three broad expression patterns. Hierarchical clustering classified the 40 candidate genes into three major expression-pattern groups. Cluster 1 includes 5 genes in MVA-pathway genes, 4 gene in MEP-pathway genes, 6 *TPS*s, and one additional gene (*Cs6g02590*). These genes generally showed highest transcript levels in HR but relatively low in CK and HR+MeJA. Cluster 2 includes 8 MEP-pathway genes but 2 MVA-pathway genes, and 4 *TPSs*. Transcript abundances of these genes were relatively low in both CK and HR but upregulated in HR+MeJA. The third cluster includes 5 genes in MVA-pathway genes and one gene in MEP-pathway genes, and 4 *TPS*s showed constitutive high levels in CK but downregulated in HR and partially restored in HR+MeJA.

Based on above analysis, four MeJA-regulated *TPS*s including *Cs3g07920*, *Cs7g18530*, *orange1.1t04360*, and *Cs2g07240* were selected for targeted qPCR validation. These four TPS genes were selected as representative pathway-related candidates rather than as a comprehensive survey of all members of the TPS gene family. Their expression patterns in response to MeJA treatment were further supported by RT–qPCR analysis (**Figure 5C**). *Cs2g07240* exhibited significant upregulation upon MeJA treatment, whereas *Cs3g07920*, *Cs7g18530*, and *orange1.1t04360* showed marked downregulation. These qPCR results are consistent with the RNA-seq data.

### 3.6 Overexpression of *Cs2g07240* in transgenic hairy roots enhanced biosynthesis of specific and non-specific terpenoid volatiles

Among the four *TPS* genes validated by qRT-PCR, *Cs2g07240* was selected for further analysis because it was the only candidate significantly induced by MeJA in both the RNA-seq and qRT-PCR analyses. Firstly, we examined its spatiotemporal expression pattern by retrieving its transcriptomic data of different tissues from the *Citrus Pan-genome to Breeding Database* (http://citrus.hzau.edu.cn/). The expression profiles showed that *Cs2g07240* is usually more strongly expressed in aboveground tissues (e.g., mature leaves, peels, pericarps and fruit calyx abscission zone) than roots. Interestingly, in fruit calyx abscission zone this gene is not only strongly expressed but also markedly upregulated in Huanglongbing (HLB) infected samples (**Figure 6A**).

**Figure 6:**
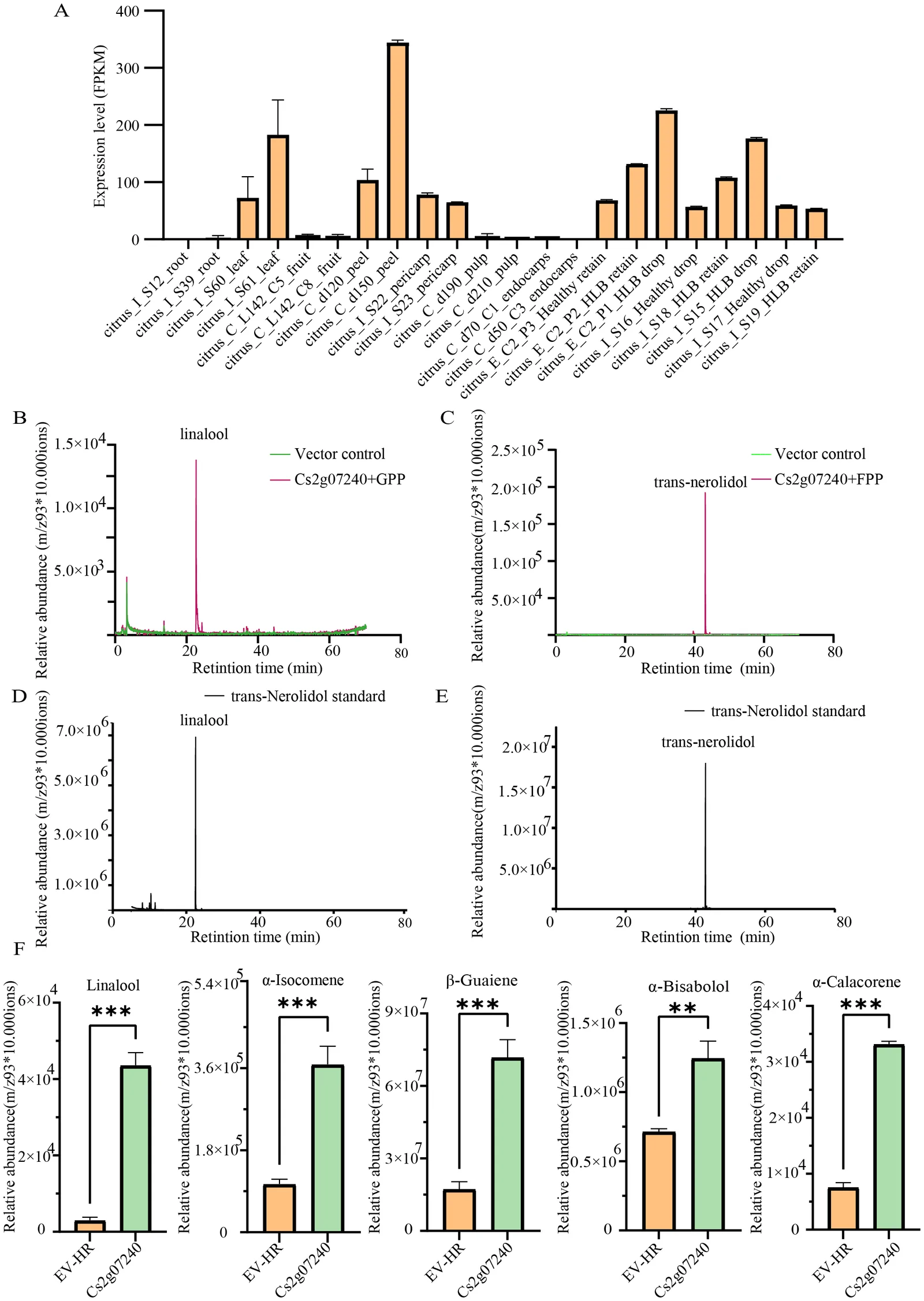
*In vivo* transgenic verification and *in vitro* enzymatic characterization of *Cs2g07240*. **(A)** Expression levels (FPKM) of *Cs2g07240* in various citrus tissues and conditions. **(B, C)** Chromatogram of product of purified *Cs2g07240* protein incubated with geranyl diphosphate (GPP) **(B)**, or with farnesyl diphosphate (FPP) **(C)**. Green lines represent empty-vector negative controls; pink lines represent *Cs2g07240* enzymatic reactions. The values are expressed as relative content based on the internal standard (IS-04) semi-quantitation method. **(D, E)** GC-MS chromatograms of authentic linalool **(D)** and trans-nerolidol **(E)** standards analyzed under the same chromatographic conditions as the reaction products. Retention times are annotated beside the corresponding peaks. Product assignments were supported by comparison of retention times with the standards and by mass spectral. **(F)** Relative extracted-ion abundances of selected terpenoid volatiles in MeJA-treated empty-vector control (EV-HR) and MeJA-treated *Cs2g07240*-overexpressing hairy roots. Relative abundances (extracted ion current at *m/z* 93 × 10^4^ ions) of linalool, α-isocomene, β-guaiene, α-bisabolol, and α-calacorene are shown. Data are presented as mean ± SD (*n* = 3). The differences between samples of the different stimulated groups were analyzed using Student’s t-test (for two-group comparisons). *, **, and ***indicate significant difference at *p* < 0.05, *p* < 0.01, and *p* < 0.001, respectively.

To investigate the biochemical function of *Cs2g07240*, we isolated its CDS and heterologously expressed it in *E. coli*. Recombinant *Cs2g07240* converted GPP to linalool and FPP to trans-nerolidol *in vitro*. The GPP-derived product and the authentic linalool standard eluted at 22.497 and 22.502 min, respectively (**Figures 6B, D**), whereas the FPP-derived product and the authentic trans-nerolidol standard eluted at 43.647 and 43.652 min, respectively (**Figures 6C, E**). The agreement in retention times, together with the corresponding mass-spectral comparisons (**Supplementary Figure S6**), supported the identification of the products as linalool and trans-nerolidol.

*Cs2g07240* was then overexpressed in citrus hairy roots via *R. rhizogenes* transformation. GC-MS analysis (**Figure 6F**) showed that overexpression of *Cs2g07240* significantly altered terpenoid volatile profiles. Linalool abundance was approximately 14.35-fold higher in *Cs2g07240*-overexpressing Carrizo citrange hairy roots than in the empty-vector control (*p* < 0.01). Surprisingly, some unexpected terpenoid volatiles including α-isocomene (3.50-fold), α-bisabolol (1.74-fold), α-calacorene (4.37-fold), and β-guaiene (4.15-fold) were also significantly upregulated. These results indicate that *Cs2g07240* overexpression in roots not only boosts linalool but also enhances a suite of other terpenoids.

### 3.7 Stable overexpression of *Cs2g07240* in sweet orange increased leaf linalool accumulation and attractiveness to *Diaphorina citri*

Given its predominant expression in aerial tissues and its induction under HLB stress, and the limitation of our hairy root system only over-expressed the gene in root, we further generated stable transgenic sweet orange plants over-expressing *Cs2g07240* in shoot. A pCAMBIA-*Cs2g07240* expression construct driven by the CaMV 35S promoter was introduced into sweet orange shoot segments via *Agrobacterium tumefaciens*-mediated transformation. Leaf tissues from three independent overexpression lines were subsequently used for volatile analysis, with plants transformed with the corresponding empty pCAMBIA vector serving as the control (**Figure 7A**). Three lines (OE_1, OE_2, and OE_3) were verified as positive transformants via comprehensive GUS, phenotypic, RT-qPCR cooperative assays (**Figures 7B–D**) and PCR (**Supplementary Figure S7B**). Leaf volatile profiling via GC-MS revealed 2.6-fold to 4.7-fold enhancements of linalool accumulation in these lines compared to EV (**Figure 7E**). However, no significant difference in trans-nerolidol detected between the transgenic and EV plants.

**Figure 7:**
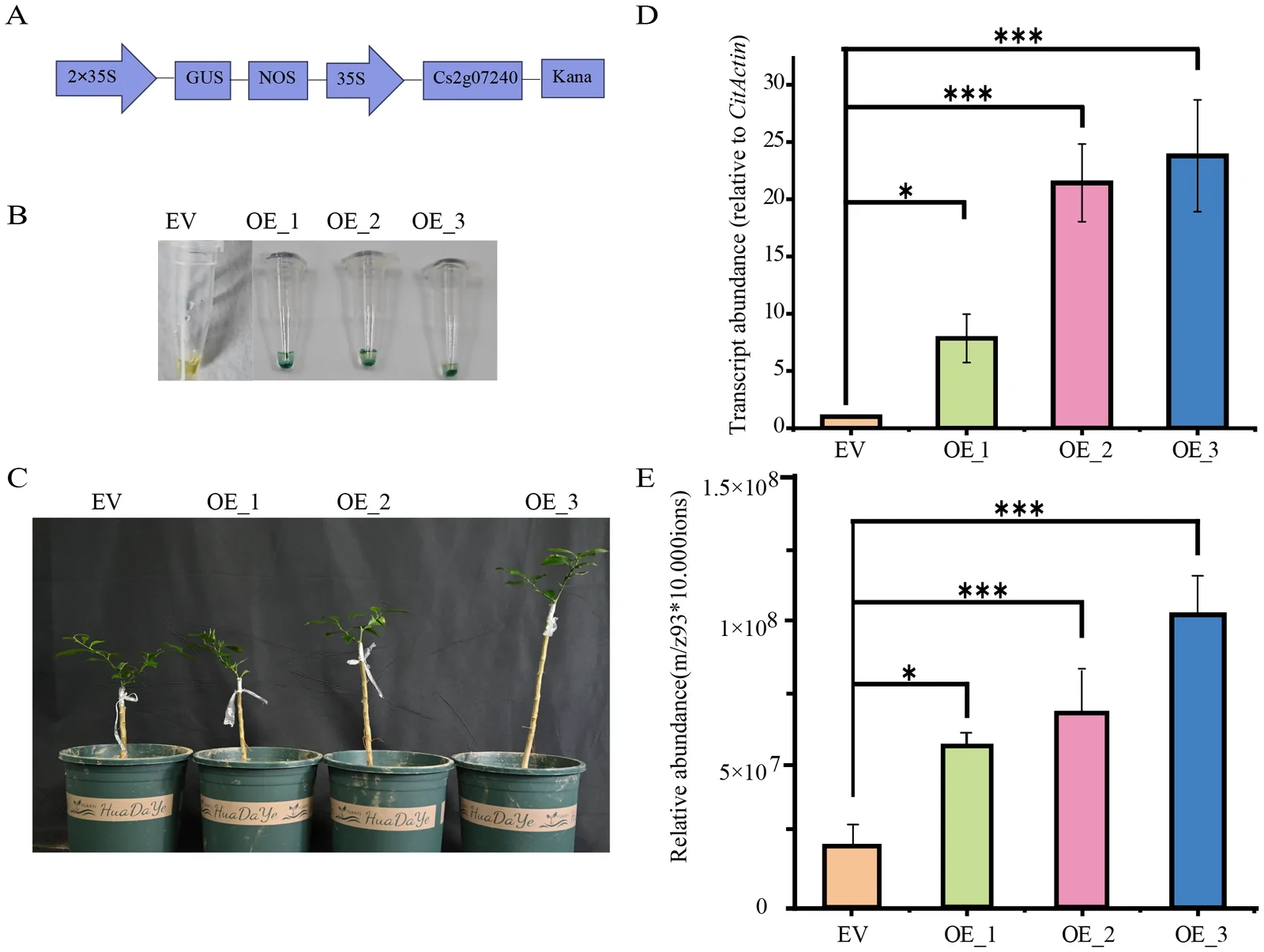
Generation and characterization of stable transgenic *Cs2g07240*-overexpressing (OE) citrus lines. **(A)** Schematic representation of the CaMV 35S promoter-driven pCAMBIA-*Cs2g07240* construct used for transformation. **(B)** GUS histochemical staining of empty-vector control (EV) and transgenic lines (OE_1, OE_2, and OE_3). **(C)** Phenotypes of the verified stable transgenic plants under greenhouse conditions. **(D)** RT-qPCR quantifying the relative transcript abundance of *Cs2g07240* in the leaves of EV and OE lines. **(E)** Relative abundance of linalool in the leaves of EV and transgenic lines determined by GC-MS. Data in **(D)** and **(E)** represent means ± SD. Differences between each overexpression line and CK were evaluated using Student’s t-test. * and *** indicate *p* < 0.05 and *p* < 0.001, respectively.

Consistent with the expression pattern observed in **Figure 6A**, we next investigated whether *Cs2g07240* responds to pathogen challenge in leaves. HLB infection significantly upregulated *Cs2g07240* expression in citrus leaves, with a 2.6-fold increase relative to healthy controls (**Figure 8A**). To elucidate the ecological consequences of this volatile modulation, the olfactory preferences of *Diaphorina citri* were evaluated using a dual-choice Y-tube olfactometer. We found that *D. citri* adults displayed a significant preference toward the leaves of *Cs2g07240*-overexpressing lines over the EV plants, with 54% to 67% of the psyllids choosing the OE lines across different transformants (**Figure 8B**). Furthermore, behavioral assays utilizing synthetic chemicals fully recapitulated this phenotype, confirming that pure linalool strongly attracted 54% to 70% of *D. citri* adults over the *n*-hexane solvent control (**Figure 8C**). Taken together, these findings demonstrate that show higher *Cs2g07240* transcript abundance in HLB-infected leaves, increased linalool accumulation in overexpressing leaves, and an olfactory preference of *D. citri* adults for these leaves and synthetic linalool.

**Figure 8:**
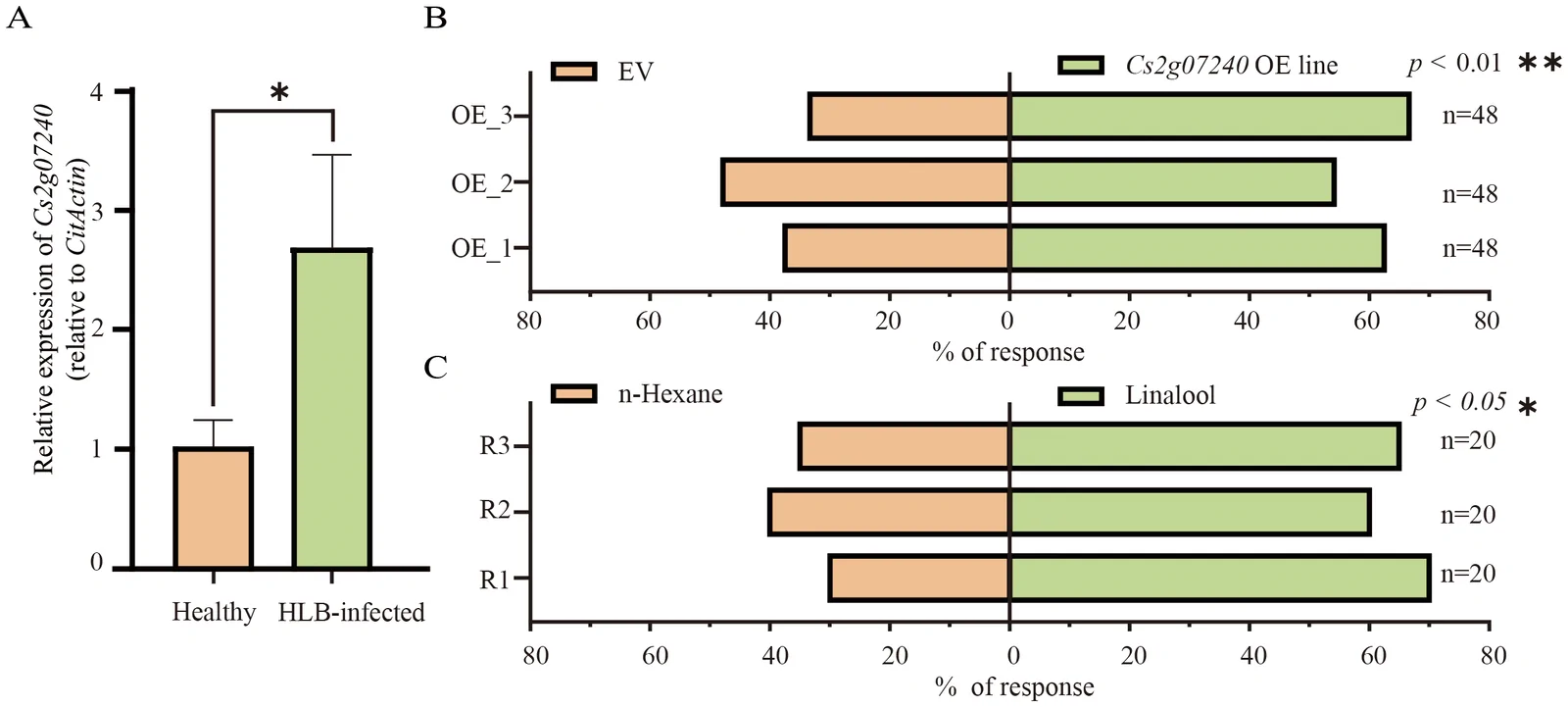
Expression of *Cs2g07240* in HLB_infected leaves and olfactory preferences of *Diaphorina citri* adults for *Cs2g07240*-overexpressing leaves and synthetic linalool. **(A)** Relative transcript abundance of *Cs2g07240* in healthy control (Healthy) and HLB-infected citrus leaves, as determined by qRT-PCR using *CitActin* as the reference gene. **(B)** Olfactory responses of *D. citri* adults to detached leaves from three independent *Cs2g07240*-overexpressing lines (OE_1, OE_2, and OE_3) versus the corresponding EV plants. Each OE-line comparison included 48 valid choices (n = 48 per line; pooled n = 144 across the three lines). The indicated *p* value was calculated from the pooled choice counts. **(C)** Olfactory responses of *D. citri* adults to synthetic linalool (1 μL/mL) versus the *n*-hexane solvent control across three independent replicates (R1–R3), with 20 valid choices per replicate. Data in **(A)** are presented as means ± SD. The difference in **(A)** was evaluated using Student’s t-test, whereas olfactory responses in **(B)** and **(C)** were evaluated using chi-square (χ²) tests. * and ** indicate *p* < 0.05 and *p* < 0.01, respectively.

## 4 Discussion

### 4.1 Terpenoids are predominant in VOCs of citrus roots

Our study demonstrated that terpenoids constitutively dominate the volatile organic compound (VOC) profiles of citrus roots, representing approximately 41%–43% of total volatiles across the CK, HR, and HR+MeJA groups. Although terpenoids also dominate the volatile profiles of citrus aerial tissues, their relative contribution in roots was lower than that previously reported for fruit peels and leaves. In fruit peel oils, they typically constitute over 90~95% of total volatiles, with D-limonene being the most abundant component (Guirong et al., 2017; Zhang et al., 2017; González-Mas et al., 2019). Similarly, terpenoids consistently comprise the vast majority (>85%) of leaf volatiles across different genotypes and developmental stages, significantly exceeding non-terpenoid compounds (Azam et al., 2013). This may be attributed to the high expression of terpenoid biosynthesis pathways in these citrus tissues, such as the genes involved in geranyl pyrophosphate (GPP, a key precursor for monoterpenes) and terpene synthase genes (e.g., GGPPS, TPS) (Alquézar et al., 2017; He et al., 2024). This phenomenon may reflect an evolutionary metabolic defense strategy in citrus, where stable production of terpenoids (broad-spectrum resistance compounds) is maintained in both above and under-ground tissues.

Although terpenoid VOCs are abundant in nearly all citrus organs, their accumulation exhibits profound spatiotemporal specialization. This distribution is coordinated by the differential regulation of terpene biosynthesis and the functional diversification of TPS genes (Alquézar et al., 2017). Specifically, young tissues prioritize the synthesis of chemical shields to compensate for their structural vulnerability. For instance, the relative abundance of limonene accounts for as much as 47.22% of total monoterpenes in young leaves of *C. reticulata* Blanco but drastically drops to a mere 4.11% in mature leaves (Azam et al., 2013). This sharp ontogenetic contrast supports a potentially important defensive role for selectively elevated limonene during the herbivore-susceptible stages of leaf development, consistent with the developmental regulation and defensive functions of plant terpenes (McConkey et al., 2000; Boege and Marquis, 2005; Gershenzon and Dudareva, 2007). Limonene accumulation may vary among plant organs, reflecting the tissue-specific biosynthesis and storage of plant volatiles (Gershenzon and Dudareva, 2007; Maffei, 2010). However, our experimental results reveal that the metabolic profile of the roots can be modulated under specific conditions. In *R. rhizogenes*-induced and MeJA-elicited hairy roots, the accumulation of limonene was slightly yet significantly upregulated compared to CK. This observation indicates that the biosynthetic pathways for limonene, while typically less active in the roots, remain responsive to exogenous elicitors and bio48tic induction within the hairy root system. Such metabolic responsiveness suggests that the root tissues possess the enzymatic potential to synthesize these monoterpenes when triggered by *R. rhizogenes*-related stress or hormonal signals, even for compounds that are predominantly associated with leaf-based defense in the natural state.

### 4.2 *R. rhizogenes* induction and MeJA stimulation strongly altered terpenoid profiles in citrus roots

We found that *R. rhizogenes* induced hairy roots exhibited altered terpenoid profiles compared to CK roots. It only slightly increased the proportion (about 0.9%) of terpenoids in the total VOCs, but it generally upregulated most of the compounds and suppressed some others. In consistent with this, *R. rhizogenes* also differentially regulated the genes involved in terpenoid biosynthesis including the MVA, MEP pathway genes and *TPS*s. The effect of *R. rhizogenes* on terpenoid biosynthesis has also been observed in other plant systems. For example, in *R. rhizogenes* induced hairy roots of *Panax ginseng* defense-oriented sesquiterpenes and triterpenes were promoted while the accumulation of growth-associated diterpenes were suppressed (Meng et al., 2022). This regulation might be mediated by the *rol* genes in the Ri plasmid in *R. rhizogenes*, which is able to influence the activities of key rate-limiting enzymes in the MVA and MEP pathways, *TPS*s and *P450*s as well as associated transcription factors, thereby enhancing the production of defense-related secondary metabolites (Tholl, 2015; Liao et al., 2016; Pu et al., 2021). The metabolic engineering potential of *R. rhizogenes*-transformed hairy roots, particularly their capacity to upregulate specialized metabolism and terpenoid biosynthesis, is well-documented (Sujatha et al., 2013; Zhang et al., 2019; Sathasivam et al., 2022). Our data reveal that *R. rhizogenes* effectively reprogramed citrus root metabolism and transcriptome, resulted in accumulation of defense-oriented terpenoids such as β-pinene and D-limonene. Mechanistically, this shift involves the coordinated and upregulation of key MVA pathway genes (HMGR, FPPS) and certain *TPS*.

This study reveals that the regulatory role of MeJA on terpenoid metabolism in hairy roots is primarily significant not in the total terpenoid production (the HR+MeJA group only showed a minor increase of approximately 0.9% relative to HR), but in driving a dramatic chemodiversity shift in the terpenoid profile. This shift highlights the precise control plants exert over resource allocation and chemical defense upon phyto-hormone regulation as possible stress responses. Transcriptome analysis further showed that MeJA treatment was associated with increased transcript levels of several genes involved in the two major terpenoid precursor biosynthesis pathways, the MVA and MEP pathways (Giri and Narasu, 2000; Wasternack and Hause, 2013). Specifically, HMGR (*Cs3g27760*) in the MVA pathway and DXR (*Cs5g05440*) in the MEP pathway showed higher transcript levels in HR+MeJA than in HR. These transcriptional changes suggest that MeJA may enhance the capacity for IPP/DMAPP precursor biosynthesis, thereby contributing to the observed changes in terpenoid accumulation. This overall activation of the MEP pathway is highly consistent with the simultaneous upregulation of monoterpenes in both the HR and HR+MeJA groups. Among the monoterpenes, (+)-limonene, α-terpinene, thujene, (*E*)-β-terpinolene, and 1,3,8-p-menthatriene showed low accumulation levels in the CK. However, their accumulation was significantly upregulated to high levels following both HR and HR+MeJA. This likely represents a broad-spectrum defense response of the roots to environmental stress and Agrobacterium induction. Consistent with literature reports (Kim et al., 2009; Akhgari et al., 2019; Nguyen et al., 2019; Sykłowska-Baranek et al., 2022), GO enrichment of the transcriptome data suggested that MeJA treatment activated the “terpenoid backbone biosynthesis” and “sesquiterpenoid and triterpenoid biosynthesis” pathways, inducing significant metabolic reprogramming. Interestingly, the transcriptomic data specifically indicated that many genes in the MEP pathway was uniquely upregulated in the hairy roots by MeJA treatments. However, the regulation of sesquiterpenes is more complex, exhibiting three types of functional differentiation. The first type, constitutive terpenes (e.g., ϵ-muurolene, α-copaene), are suppressed under strong MeJA signals (e.g., suppression of the *Cs7g18530* gene), suggesting the plant is executing a resource allocation trade-off to direct carbon flux toward more targeted products; the second type, *R. rhizosphere* adaptation terpenes (e.g., α-muurolene, *cis*-α-bergamotene), are highly expressed in both HR and HR+MeJA groups, reflecting adaptation to the rhizosphere environment; and the third type, specifically induced terpenes (e.g., cedrene, β-sabinene), only spike in the HR+MeJA group. This metabolic outcome perfectly aligns with the “HR+MeJA specific high expression” pattern exhibited by multiple TPS (e.g., *Cs1g04360* and *orange1.1t04775)*. These specific products are highly likely to be the core semiochemicals activated by the jasmonate signal, playing critical direct or indirect defense roles in the terpene-insect-plant tripartite interaction.

Crucially, while elicitation transiently boosts both the MEP and MVA pathways, it is vital to note that as a heterotrophic, non-photosynthetic rhizosphere organ, the citrus hairy root system inherently retains a massive, dominant metabolic background channeled through the cytosolic MVA pathway. This localized pool of cytosolic precursors establishes an optimized biochemical environment that heavily predisposes transgenic hairy roots toward amplified sesquiterpene cyclization pathways when further engineered downstream.

### 4.3 Overexpressing of *Cs2g07240* in citrus shoot enhanced biosynthesis of terpenoids more than targeted compounds

Overexpression of the orthologous *Cs2g07240*, the orthologous gene of a characterized linalool synthase gene *CuSTS3–2* from *Citrus unshiu* Marc. in citrange hairy roots indeed boosted the linalool accumulation to as much as14.35-fold the CK. In *C. unshiu*, *CuSTS3–2* was predominantly expressed in flowers and fruit peels. While *CuSTS3–2* was reported to only accept GPP to produce linalool, its homologous *CuSTS4* accepts both GPP for linalool and FPP for nerolidol (Pazouki and Niinemets, 2016). In our *in vitro* enzymatic assays, recombinant *Cs2g07240* is indeed capable of converting GPP to linalool and FPP to *trans*-nerolidol. Intriguingly, when overexpressed *in vivo* within the transgenic hairy root system, trans-nerolidol was completely undetectable; instead, it induced a massive surge in alternative cyclic sesquiterpenes, including α-isocomene, α-bisabolol, α-calacorene, and particularly β-guaiene, were significantly higher in the *Cs2g07240*-overexpressing roots than in the empty-vector control, with β-guaiene showing an approximately 4.15-fold increase (**Figure 6F**). This apparent discrepancy between *in vitro* catalytic potential and *in vivo* output in hairy roots highlights the critical role of localized substrate availability and plant cell microenvironments. As described in section 4.2, the heterotrophic hairy root system natively maintains an extremely active cytosolic MVA pathway that floods the cell with FPP. When *Cs2g07240* was overexpressed in the root environment, a systemic expansion across broader endogenous terpenoid networks was captured instead of a proportional release of free *trans*-nerolidol. Two plausible mechanisms may account for this collateral metabolic reconfiguration: either the synthesized *trans*-nerolidol serves as a transient intermediate that is rapidly channeled into and consumed by downstream endogenous modifying enzymes (Aharoni et al., 2003; Davidovich-Rikanati et al., 2007), or the over accumulated linalool acts as an intracellular signaling molecule that retroactively triggers a transcriptional cascade of wide-spectrum indigenous *TPS* genes (Taniguchi et al., 2014; Riedlmeier et al., 2017). To fully validate this substrate-dependent plasticity *in planta*, we successfully generated stable transgenic citrus plants overexpressing *Cs2g07240*. Remarkably, in the stable overexpressing lines (OE_1, OE_2, and OE_3), the volatile profile of the green leaf tissue—which possesses an abundant, chloroplast-localized MEP pathway supplying GPP (C_10_)—shifted entirely to favor the specific and significant accumulation of linalool (**Figure 7E**). The phenomenon where single *TPS* overexpression dramatically alters non-targeted specialized metabolites can be further interpreted through multiple intrinsic regulatory mechanisms. One reasonable explanation is that the direct products of our ectopically expressed genes could be further converted or modified into other related downstream metabolites by the indigenous biochemical environment of the citrus host cells. Another intriguing possibility is that some of these heavily induced terpenoids or intermediate volatiles could function as signaling regulators of the general terpenoid biosynthesis pathways. Therefore, though only a single *TPS* was overexpressed in the transgenic roots, some other indigenous *TPS* nodes could be subsequently trans-activated by the feedback products of the expressed genes. Similar to our discovery, ectopic engineering of the *Arabidopsis thaliana* (*E*)-β-caryophyllene synthase *TPS21* in citrus also highly disturbed the accumulation and emission of many mono- and sesquiterpenoid volatiles other than (*E*)-β-caryophyllene (Alquézar et al., 2021). Taken together, ectopic overexpression of *CsTPSs* in citrus hairy roots and stable leaves brought consequences far more comprehensive than the isolated enhancements of the responsive products directly synthesized by the expressed genes, orchestrating an expanded chemical defense system that effectively modulates pest behavioral responses. Importantly, this volatile reconfiguration extends to host–pathogen–vector interactions, strongly supporting the “Pathogen Manipulation Hypothesis” (Mauck et al., 2010). We found that HLB infection significantly induced *Cs2g07240* expression by 2.6-fold, closely mirroring the robust behavioral attraction of *D. citri* adults toward *Cs2g07240*-overexpressing leaves and synthetic linalool. This indicates that the HLB pathogen evolved to subvert host plant volatile networks via *Cs2g07240* activation, turning the citrus host into an alluring chemical beacon for its insect vector to maximize transmission efficiency (Alquézar et al., 2021). Consequently, disrupting this pathogen-hijacked olfactory signaling pathway could offer a targeted approach for preventing HLB transmission. This contrast between leaves and hairy roots suggests that the chemical phenotype associated with *Cs2g07240* may depend on the metabolic context. However, the root experiments used Carrizo citrange, whereas stable transformation used sweet orange; differences in genetic background, transformation system, and treatment conditions therefore limit the attribution of these differences solely to tissue identity. The findings may not apply uniformly to other citrus species or genotypes, and further studies under matched conditions are needed to assess their broader applicability.

## 5 Conclusion

In conclusion, we found that citrus roots accumulate a large set of volatile organic compounds, particularly terpenoids. In *R. rhizogenes*-induced citrus hairy roots, these terpenoids and the corresponding biosynthetic genes were differentially regulated by *R. rhizogenes* transformation and were further modulated by exogenous MeJA. Overexpression of a MeJA-regulated linalool/nerolidol synthase gene, *Cs2g07240*, separately increased linalool accumulation in hairy roots and in leaves of stable transgenic sweet orange plants. Moreover, *Cs2g07240* was HLB responsive, and transgenic shoots overexpressing this gene were more attractive to adults of *D. citri*, the insect vector of HLB. Together, this study unveils tissue-specific terpene metabolic networks in citrus and identifies a key synthase with divergent catalytic outputs between *in vitro* and *in planta* systems, providing a basis for understanding volatile-mediated ecological interactions.

## Data Availability

The datasets presented in this study can be found in online repositories. The names of the repository/repositories and accession number(s) can be found in the article/**Supplementary Material**.

## References

[B1] Aharoni A. Giri A. P. Deuerlein S. Griepink F. De Kogel W. J. Verstappen F. W. A. . (2003). Terpenoid metabolism in wild-type and transgenic *Arabidopsis* plants. Plant Cell 15, 2866–2884. doi: 10.1105/tpc.016253 14630967 PMC282818

[B2] Akhgari A. Laakso I. Maaheimo H. Choi Y. H. Seppänen-Laakso T. Oksman-Caldentey K.-M. . (2019). Methyljasmonate elicitation increases terpenoid indole alkaloid accumulation in *Rhazya stricta* hairy root cultures. Plants 8, 534. doi: 10.3390/plants8120534 31766620 PMC6963348

[B3] Alquézar B. Rodríguez A. De La Peña M. Peña L. (2017). Genomic analysis of terpene synthase family and functional characterization of seven sesquiterpene synthases from *Citrus sinensis*. Front. Plant Sci. 8. doi: 10.3389/fpls.2017.01481 28883829 PMC5573811

[B4] Alquézar B. Volpe H. X. L. Magnani R. F. De Miranda M. P. Santos M. A. Marques V. V. . (2021). Engineered orange ectopically expressing the *Arabidopsis* β-caryophyllene synthase is not attractive to *Diaphorina citri*, the vector of the bacterial pathogen associated to Huanglongbing. Front. Plant Sci. 12, 641457. doi: 10.3389/fpls.2021.641457 33763099 PMC7982956

[B5] Alsabte A. Ahmed Q. H. Kayahan A. Karaca I. (2022). Effects of volatile organic compounds (VOCs) emitted by citrus infested with *Aonidiella aurantii* on the predator *Rhyzobius lophanthae* attraction. Phytoparasitica 50, 645–653. doi: 10.1007/s12600-022-00978-430311153

[B6] Anand A. Jayaramaiah R. H. Beedkar S. D. Dholakia B. B. Lavhale S. G. Punekar S. A. . (2019). Terpene profiling, transcriptome analysis and characterization of *cis*-terpineol synthase from *Ocimum*. Physiol. Mol. Biol. Plants 25, 47–57. doi: 10.1007/s12298-018-0612-6 30804629 PMC6352525

[B7] Azam M. Jiang Q. Zhang B. Xu C. J. Chen K. S. (2013). Citrus leaf volatiles as affected by developmental stage and genetic type. Int. J. Mol. Sci. 14, 17744–17766. doi: 10.3390/ijms140917744 23994837 PMC3794751

[B8] Bittner A. Ciesla A. Gruden K. Lukan T. Mahmud S. Teige M. . (2022). Organelles and phytohormones: a network of interactions in plant stress responses. J. Exp. Bot. 73, 7165–7181. doi: 10.1093/jxb/erac384 36169618 PMC9675595

[B9] Boege K. Marquis R. J. (2005). Facing herbivory as you grow up: the ontogeny of resistance in plants. Trends Ecol. Evol. 20, 441–448. doi: 10.1016/j.tree.2005.05.001 16701415

[B10] Chen F. Tholl D. Bohlmann J. Pichersky E. (2011). The family of terpene synthases in plants: a mid-size family of genes for specialized metabolism that is highly diversified throughout the kingdom. Plant J. 66, 212–229. doi: 10.1111/j.1365-313X.2011.04520.x 21443633

[B11] Davidovich-Rikanati R. Sitrit Y. Tadmor Y. Iijima Y. Bilenko N. Bar E. . (2007). Enrichment of tomato flavor by diversion of the early plastidial terpenoid pathway. Nat. Biotechnol. 25, 899–901. doi: 10.1038/nbt1312 17592476

[B12] Dornelas M. C. Mazzafera P. (2007). A genomic approach to characterization of the *Citrus* terpene synthase gene family. Genet. Mol. Biol. 30, 832–840. doi: 10.1590/S1415-4757200700050001141099703

[B13] Dosoky N. S. Setzer W. N. (2018). Biological activities and safety of *Citrus* spp. essential oils. Int. J. Mol. Sci. 19, 1966. doi: 10.3390/ijms19071966 29976894 PMC6073409

[B14] Gajera H. P. Hirpara D. G. Katakpara Z. A. Patel S. V. Golakiya B. A. (2016). Molecular evolution and phylogenetic analysis of biocontrol genes acquired from SCoT polymorphism of mycoparasitic *Trichoderma koningii* inhibiting phytopathogen *Rhizoctonia solani* Kuhn. Infect. Genet. Evol. 45, 383–392. doi: 10.1016/j.meegid.2016.09.026 27720889

[B15] Georgiev M. I. Ludwig-Müller J. Bley T. (2010). “ Hairy root culture: copying nature in new bioprocesses,” in Medicinal Plant Biotechnology ( CABI, Wallingford UK), 156–175.

[B16] Gershenzon J. Dudareva N. (2007). The function of terpene natural products in the natural world. Nat. Chem. Biol. 3, 408–414. doi: 10.1038/nchembio.2007.5 17576428

[B18] Gfeller V. Huber M. Förster C. Huang W. Köllner T. G. Erb M. (2019). Root volatiles in plant–plant interactions I: high root sesquiterpene release is associated with increased germination and growth of plant neighbours. Plant Cell Environ. 42, 1950–1963. doi: 10.1111/pce.13532 30737807 PMC6850102

[B19] Giri A. Narasu M. L. (2000). Transgenic hairy roots: recent trends and applications. Biotechnol. Adv. 18, 1–22. doi: 10.1016/S0734-9750(99)00016-6 14538116

[B20] González-Mas M. C. Rambla J. L. López-Gresa M. P. Blázquez M. A. Granell A. (2019). Volatile compounds in *Citrus* essential oils: a comprehensive review. Front. Plant Sci. 10, 12. doi: 10.3389/fpls.2019.00012 30804951 PMC6370709

[B21] Grover S. Aggarwal P. Kumar A. Kaur S. Yadav R. Babbar N. (2025). Utilizing *Citrus* peel waste: a review of essential oil extraction, characterization, and food-industry potential. Biomass Convers. Biorefin. 15, 5043–5064. doi: 10.1007/s13399-024-05382-y30311153

[B22] Guirong F. Jiao X. Lili D. Kaifang Z. Shixiang Y. (2017). Progress on terpenoid volatiles in *Citrus* fruits. Food. Mach. 33, 200–204. doi: 10.13652/j.issn.1003-5788.2017.10.043

[B23] Häkkinen S. T. Oksman-Caldentey K.-M. (2018). “ Progress and prospects of hairy root research,” in Hairy Roots: An Effective Tool of Plant Biotechnology. Eds. Srivastava V. Mehrotra S. Mishra S. ( Springer, Singapore), 3–19.

[B24] He J. Fandino R. A. Halitschke R. Luck K. Köllner T. G. Murdock M. H. . (2019). An unbiased approach elucidates variation in (S)-(+)-linalool, a context-specific mediator of a tri-trophic interaction in wild tobacco. Proc. Natl. Acad. Sci. U.S.A. 116, 14651–14660. doi: 10.1073/pnas.1818585116 31262827 PMC6642400

[B25] He J. Halitschke R. Schuman M. C. Baldwin I. T. (2021). Light dominates the diurnal emissions of herbivore-induced volatiles in wild tobacco. BMC Plant Biol. 21, 401. doi: 10.1186/s12870-021-03179-z 34461825 PMC8404343

[B26] He J. X. Qin Z. Y. Liu K. X. Li X. Y. Kou Y. M. Jin Z. H. . (2024). Volatile metabolomics and transcriptomics analyses provide insights into the mechanism of volatile changes during fruit development of 'Ehime 38' (*Citrus reticulata*) and its bud mutant. Front. Plant Sci. 15, 1430204. doi: 10.3389/fpls.2024.1430204 38984161 PMC11231921

[B27] Huang M. Y. Valim M. F. Feng S. Reuss L. Yao L. X. Gmitter F. . (2017). Characterization of the major aroma-active compounds in peel oil of an HLB-tolerant mandarin hybrid using aroma extraction dilution analysis and gas chromatography-mass spectrometry/olfactometry. Chemosens. Percept. 10, 161–169. doi: 10.1007/s12078-017-9221-y30311153

[B28] Irigoyen S. Ramasamy M. Pant S. Niraula P. Bedre R. Gurung M. . (2020). Plant hairy roots enable high throughput identification of antimicrobials against *Candidatus* Liberibacter spp. Nat. Commun. 11, 5802. doi: 10.1038/s41467-020-19631-x 33199718 PMC7669877

[B29] Javed S. Mahmood Z. Khan K. M. Sarker S. D. Javaid A. Khan I. H. . (2021). Lupeol acetate as a potent antifungal compound against opportunistic human and phytopathogenic mold *Macrophomina phaseolina*. Sci. Rep. 11, 8417. doi: 10.1038/s41598-021-87725-7 33875698 PMC8055904

[B30] Jones S. E. Killiny N. (2021). Influence of rootstock on the leaf volatile organic compounds of citrus scion is more pronounced after the infestation with *Diaphorina citri*. Plants 10, 2422. doi: 10.3390/plants10112422 34834785 PMC8623621

[B31] Kim O. T. Bang K. H. Kim Y. C. Hyun D. Y. Kim M. Y. Cha S. W. (2009). Upregulation of ginsenoside and gene expression related to triterpene biosynthesis in ginseng hairy root cultures elicited by methyl jasmonate. Plant Cell Tissue Organ Cult. 98, 25–33. doi: 10.1007/s11240-009-9535-930311153

[B32] Liao P. Hemmerlin A. Bach T. J. Chye M. L. (2016). The potential of the mevalonate pathway for enhanced isoprenoid production. Biotechnol. Adv. 34, 697–713. doi: 10.1016/j.biotechadv.2016.03.005 26995109

[B36] Maffei M. E. (2010). Sites of synthesis, biochemistry and functional role of plant volatiles. S. Afr. J. Bot. 76, 612–631. doi: 10.1016/j.sajb.2010.03.00342574925

[B33] Magnani R. F. Volpe H. X. L. Luvizotto R. A. G. Mulinari T. A. Agostini T. T. Bastos J. K. . (2025). α-Copaene is a potent repellent against the Asian citrus psyllid *Diaphorina citri*. Sci. Rep. 15, 3564. doi: 10.1038/s41598-025-86369-1 39875401 PMC11775201

[B34] Maleki F. A. Seidl-Adams I. Felton G. W. Kersch-Becker M. F. Tumlinson J. H. (2024). Stomata: gatekeepers of uptake and defense signaling by green leaf volatiles in maize. J. Exp. Bot. 75, 6872–6887. doi: 10.1093/jxb/erae401 39397371

[B35] Mauck K. E. De Moraes C. M. Mescher M. C. (2010). Deceptive chemical signals induced by a plant virus attract insect vectors to inferior hosts. PNAS 107, 3600–3605. doi: 10.1073/pnas.0907191107 20133719 PMC2840436

[B40] McConkey M. E. Gershenzon J. Croteau R. B. (2000). Developmental regulation of monoterpene biosynthesis in the glandular trichomes of peppermint. Plant Physiol. 122, 215–223. doi: 10.1104/pp.122.1.215 10631265 PMC58860

[B37] Meng X. Zhang T. Chen C. Li Q. Liu J. (2022). Regulatory network of ginsenoside biosynthesis under Ro stress in the hairy roots of *Panax ginseng* revealed by RNA sequencing. Front. Bioeng. Biotechnol. 10, 1006386. doi: 10.3389/fbioe.2022.1006386 36394021 PMC9659575

[B38] Mirowski P. D. Bueno G. Rodrigues V. E. Barros T. F. Da Costa A. G. Jr. Yoshida N. C. . (2023). Chemical composition and evaluation of antibacterial, antibiofilm, and mutagenic potentials of a propolis sample from the Atlantic Forest of Midwest Brazil. Chem. Biodivers. 20, e202301238. doi: 10.1002/cbdv.202301238 37769153

[B39] Mohammed K. Agarwal M. Li B. B. Newman J. Liu T. Ren Y. L. (2020). Evaluation of d-limonene and β-ocimene as attractants of *Aphytis melinus* (Hymenoptera: Aphelinidae), a parasitoid of *Aonidiella aurantii* (Hemiptera: Diaspididae) on *Citrus* spp. Insects 11, 44. doi: 10.3390/insects11010044 31936196 PMC7022879

[B41] Nguyen K. V. Pongkitwitoon B. Pathomwichaiwat T. Viboonjun U. Prathanturarug S. (2019). Effects of methyl jasmonate on the growth and triterpenoid production of diploid and tetraploid *Centella asiatica* (L.) Urb. hairy root cultures. Sci. Rep. 9, 18665. doi: 10.1038/s41598-019-54460-z 31822691 PMC6904556

[B42] Pandey V. K. Tripathi A. Srivastava S. Dar A. H. Singh R. Farooqui A. . (2023). Exploiting the bioactive properties of essential oils and their potential applications in food industry. Food Sci. Biotechnol. 32, 885–902. doi: 10.1007/s10068-023-01287-0 37123062 PMC10130317

[B43] Pazouki L. Niinemets Ü. (2016). Multi-substrate terpene synthases: their occurrence and physiological significance. Front. Plant Sci. 7, 1019. doi: 10.3389/fpls.2016.01019 27462341 PMC4940680

[B44] Pena L. Cervera M. Juárez J. Ortega C. Pina J. Durán-Vila N. . (1995). High efficiency *Agrobacterium*-mediated transformation and regeneration of citrus. Plant Sci. 104, 183–191. doi: 10.1016/0168-9452(94)04021-8

[B45] Pérez-Hedo M. Gallego-Giraldo C. Forner-Giner M. A. Ortells-Fabra R. Urbaneja A. (2024). Plant volatile-triggered defense in citrus against biotic stressors. Front. Plant Sci. 15, 1425364. doi: 10.3389/fpls.2024.1425364 39049855 PMC11266131

[B46] Pu X. J. Dong X. M. Li Q. Chen Z. X. Liu L. (2021). An update on the function and regulation of methylerythritol phosphate and mevalonate pathways and their evolutionary dynamics. J. Integr. Plant Biol. 63, 1211–1226. doi: 10.1111/jipb.13076 33538411

[B47] Qiao Y. J. Gu C. Z. Zhu H. T. Wang D. Zhang M. Y. Zhang Y. X. . (2020). Allelochemicals of *Panax notoginseng* and their effects on various plants and rhizosphere microorganisms. Plant Divers. 42, 323–333. doi: 10.1016/j.pld.2020.04.003 33134615 PMC7584786

[B48] Qin Y. Y. Gong Y. Kong S. Y. Wan Z. Y. Liu J. Q. Xing K. . (2024). Aerial signaling by plant-associated *Streptomyces setonii* WY228 regulates plant growth and enhances salt stress tolerance. Microbiol. Res. 286, 127823. doi: 10.1016/j.micres.2024.127823 38959523

[B49] Reisenman C. E. Riffell J. A. Duffy K. Pesque A. Mikles D. Goodwin B. (2013). Species-specific effects of herbivory on the oviposition behavior of the moth *Manduca sexta*. J. Chem. Ecol. 39, 76–89. doi: 10.1007/s10886-012-0228-1 23274850

[B50] Riedlmeier M. Ghirardo A. Wenig M. Knappe C. Koch K. Georgii E. . (2017). Monoterpenes support systemic acquired resistance within and between plants. Plant Cell 29, 1440–1459. doi: 10.1105/tpc.16.00898 28536145 PMC5502447

[B51] Robert-Seilaniantz A. Grant M. Jones J. D. G. (2011). Hormone crosstalk in plant disease and defense: more than just jasmonate-salicylate antagonism. Annu. Rev. Phytopathol. 49, 317–343. doi: 10.1146/annurev-phyto-073009-114447 21663438

[B52] Rodríguez A. Shimada T. Cervera M. Alquézar B. Gadea J. Gómez-Cadenas A. . (2014). Terpene down-regulation triggers defense responses in transgenic orange leading to resistance against fungal pathogens. Plant Physiol. 164, 321–339. doi: 10.1104/pp.113.224279 24192451 PMC3875811

[B53] Saini R. K. Ranjit A. Sharma K. Prasad P. Shang X. Gowda K. G. M. . (2022). Bioactive compounds of citrus fruits: a review of composition and health benefits of carotenoids, flavonoids, limonoids, and terpenes. Antioxidants 11, 239. doi: 10.3390/antiox11020239 35204122 PMC8868476

[B54] Sathasivam R. Choi M. Radhakrishnan R. Kwon H. Yoon J. Yang S. H. . (2022). Effects of various *Agrobacterium rhizogenes* strains on hairy root induction and analyses of primary and secondary metabolites in *Ocimum basilicum*. Front. Plant Sci. 13, 983776. doi: 10.3389/fpls.2022.983776 36325544 PMC9619037

[B55] Sevón N. Oksman-Caldentey K.-M. (2002). *Agrobacterium rhizogenes*-mediated transformation: root cultures as a source of alkaloids. Planta Med. 68, 859–868. doi: 10.1055/s-2002-34924 12391546

[B56] Sharon-Asa L. Shalit M. Frydman A. Bar E. Holland D. Or E. . (2003). Citrus fruit flavor and aroma biosynthesis: isolation, functional characterization, and developmental regulation of *CsTPS1*, a key gene in the production of the sesquiterpene aroma compound valencene. Plant J. 36, 664–674. doi: 10.1046/j.1365-313X.2003.01910.x 14617067

[B57] Shimada T. Endo T. Fujii H. Rodríguez A. Peña L. Omura M. (2014). Characterization of three linalool synthase genes from *Citrus unshiu* Marc. and analysis of linalool-mediated resistance against *Xanthomonas citri* subsp. *citri* and *Penicilium italicum* in citrus leaves and fruits. Plant Sci. 229, 154–166. doi: 10.1016/j.plantsci.2014.09.008 25443842

[B58] Sujatha G. Zdravković-Korać S. Ćalić D. Flamini G. Kumari B. R. (2013). High-efficiency *Agrobacterium rhizogenes*-mediated genetic transformation in *Artemisia vulgaris*: hairy root production and essential oil analysis. Ind. Crops Prod. 44, 643–652. doi: 10.1016/j.indcrop.2012.09.00742574925

[B59] Sykłowska-Baranek K. Kamińska M. Pączkowski C. Pietrosiuk A. Szakiel A. (2022). Metabolic modifications in terpenoid and steroid pathways triggered by methyl jasmonate in *Taxus*× *media* hairy roots. Plants 11, 1120. doi: 10.3390/plants11091120 35567120 PMC9100385

[B60] Taniguchi S. Hosokawa-Shinonaga Y. Tamaoki D. Yamada S. Akimitsu K. Gomi K. (2014). Jasmonate induction of the monoterpene linalool confers resistance to rice bacterial blight and its biosynthesis is regulated by JAZ protein in rice. Plant Cell Environ. 37, 451–461. doi: 10.1111/pce.12169 23889289

[B61] Tholl D. (2015). “ Biosynthesis and biological functions of terpenoids in plants,” in Biotechnology of Isoprenoids, vol. 148 . Eds. Schrader J. Bohlmann J. ( Springer, Cham), 63–106. 10.1007/10_2014_295 25583224

[B62] Upadhyay R. Saini R. Shukla P. Tiwari K. (2025). Role of secondary metabolites in plant defense mechanisms: a molecular and biotechnological insights. Phytochem. Rev. 24, 953–983. doi: 10.1007/s11101-024-09976-230311153

[B63] Vranová E. Coman D. Gruissem W. (2013). Network analysis of the MVA and MEP pathways for isoprenoid synthesis. Annu. Rev. Plant Biol. 64, 665–700. doi: 10.1146/annurev-arplant-050312-120116 23451776

[B64] Wang J. Yu L. Zhao J. Fu S. Mei Y. Lou B. . (2024). ClBeclin1 positively regulates citrus defence against citrus yellow vein clearing virus through mediating autophagy-dependent degradation of ClAPX1. Mol. Plant Pathol. 25, e70041. doi: 10.1111/mpp.70041 39658820 PMC11631719

[B65] Wasternack C. Hause B. (2013). Jasmonates: biosynthesis, perception, signal transduction and action in plant stress response, growth and development. An update to the 2007 review in *Annals of Botany*. Ann. Bot. 111, 1021–1058. doi: 10.1093/aob/mct067 23558912 PMC3662512

[B66] Wei C. Y. Yang H. Z. Li R. R. Su Y. K. Li X. Z. Zhang B. (2024). Functional genomics reveals functions of terpene synthases for volatile terpene formation in peach. Food Qual. Saf. 8, fyae027. doi: 10.1093/fqsafe/fyae02740388063

[B67] Zhang B. Huo Y. Zhang J. Zhang X. Zhu C. (2019). *Agrobacterium rhizogenes*-mediated RNAi of *Tripterygium wilfordii* and application for functional study of terpenoid biosynthesis pathway genes. Ind. Crops Prod. 139, 111509. doi: 10.1016/j.indcrop.2019.11150942574925

[B68] Zhang H. P. Xie Y. X. Liu C. H. Chen S. L. Hu S. S. Xie Z. Z. . (2017). Comprehensive comparative analysis of volatile compounds in citrus fruits of different species. Food Chem. 230, 316–326. doi: 10.1016/j.foodchem.2017.03.040 28407917

[B69] Zheng C. Chen J. P. Wang X. W. Li P. (2025). Reactive oxygen species in plants: metabolism, signaling, and oxidative modifications. Antioxidants 14, 617. doi: 10.3390/antiox14060617 40563252 PMC12190107

