# Supplementary material for: Integrated metabolomic and transcriptomic profiling uncovers a citrus terpene synthase with in vivo–in vitro catalytic divergence and psyllid-attracting ecological function
Source: Front Plant Sci. 2026 Sep 29;17:1911599. doi: 10.3389/fpls.2026.1911599 (PMC13658172; doi:10.3389/fpls.2026.1911599)
Supplement: Supplementary file 1 [file DataSheet1.pdf]

supplemental figure

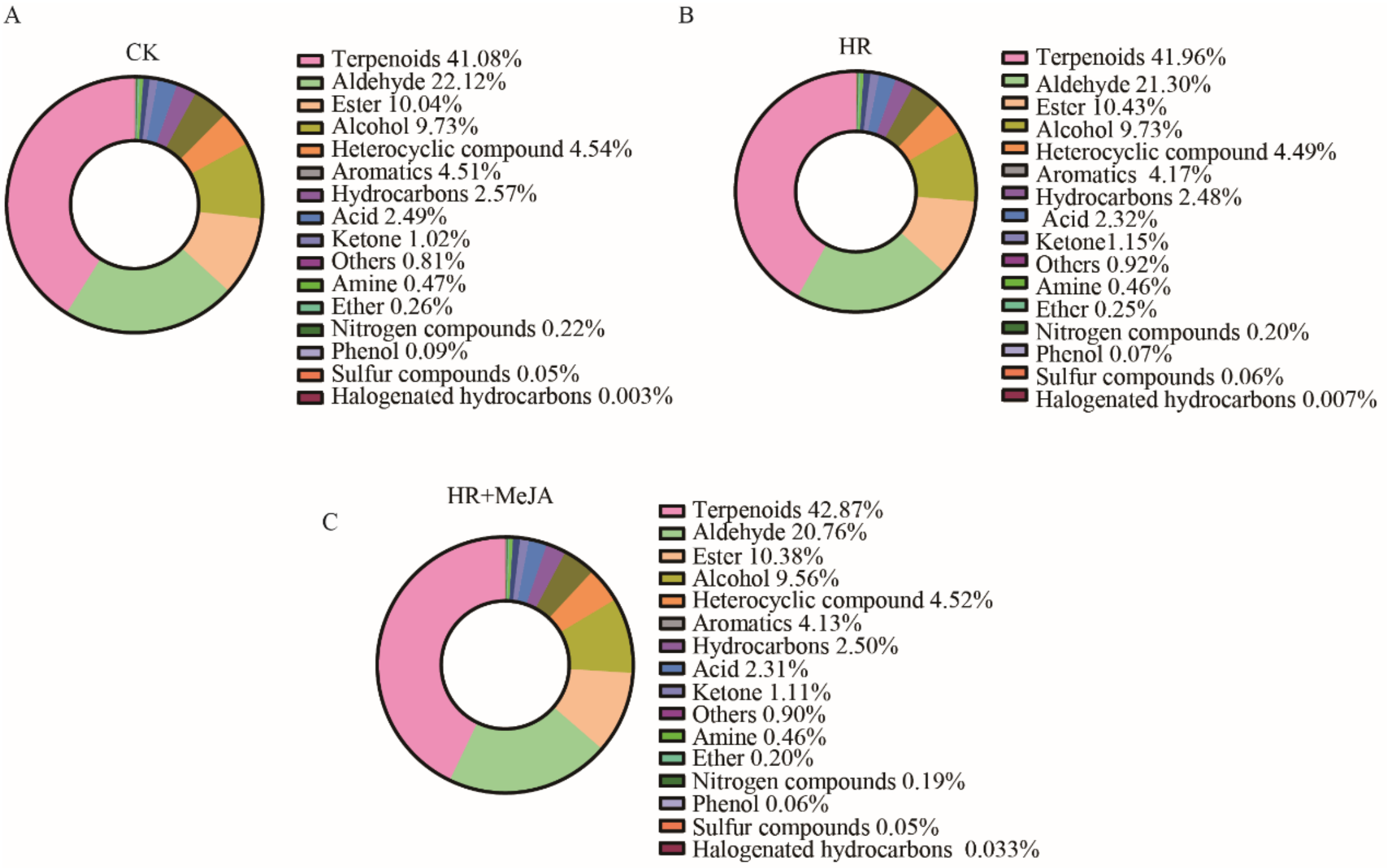

Fig. S1 General classification and proportion of the volatile organic compounds (VOCs) detected in (A) control (CK), (B) HR+ methyl jasmonate (MeJA), and (C) hairy roots (HR) samples. For each VOC, the mean GC-MS signal abundance was calculated across replicate samples within each group. The resulting mean abundances were then summed within each chemical class. Class percentages were calculated by dividing each class sum by the sum for all VOCs included in that group's analysis and multiplying by 100.

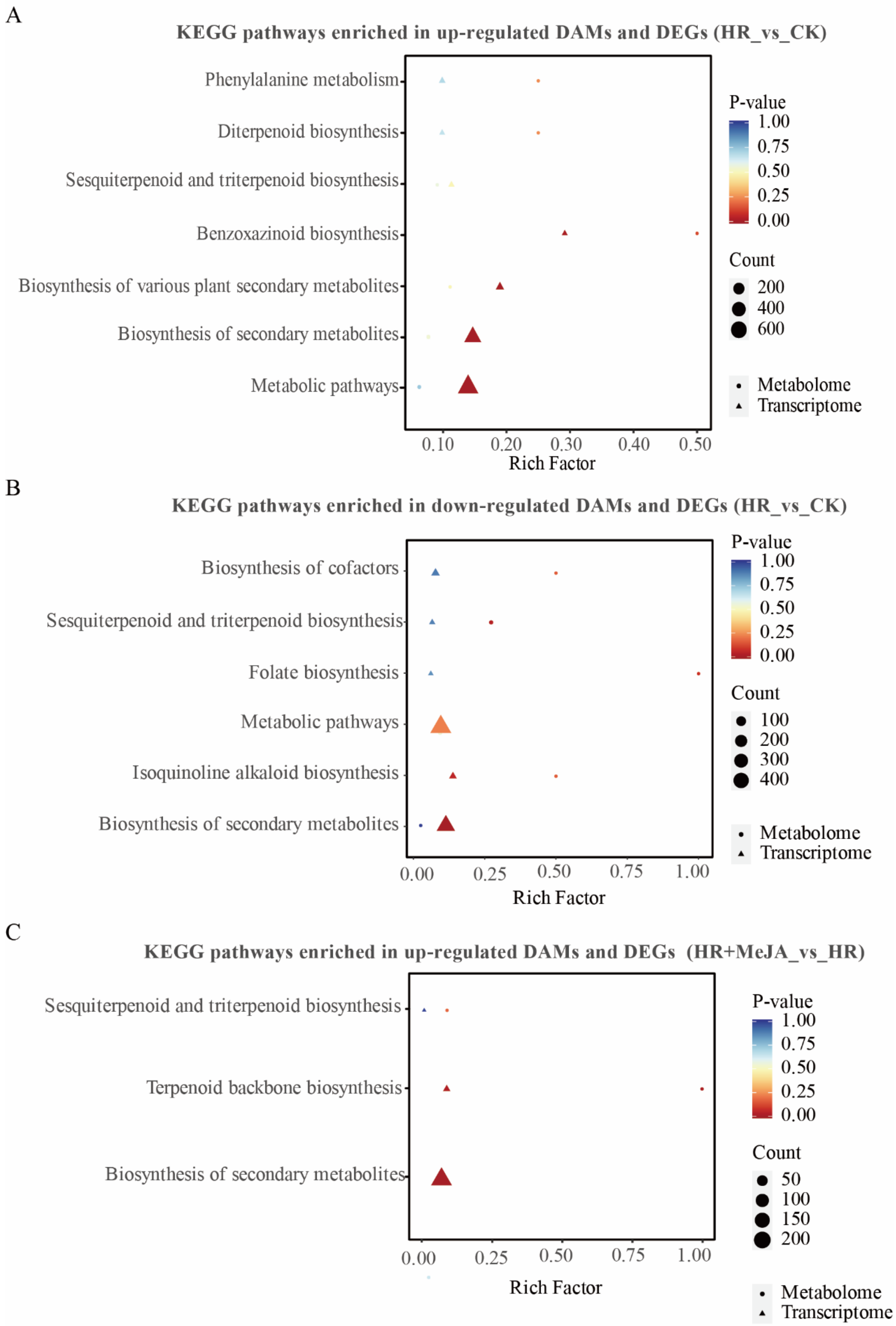

Fig. S2. KEGG pathway enrichment of upregulated and downregulated genes and metabolites in the integrated transcriptomic and metabolomic analysis. (A, B) Upregulated (A) and downregulated (B) DEGs and DAMs in HR relative to CK. (C) Upregulated DEGs and DAMs in MeJA+ HR relative to HR. Triangles and circles represent transcriptomic and metabolomic data, respectively.

Symbol size indicates the number of DEGs or DAMs mapped to each pathway, and color represents the P-value. The rich factor is the ratio of the number of DEGs or DAMs mapped to a pathway to the total number of background genes or metabolites annotated to that pathway, respectively. MeJA, methyl jasmonate.

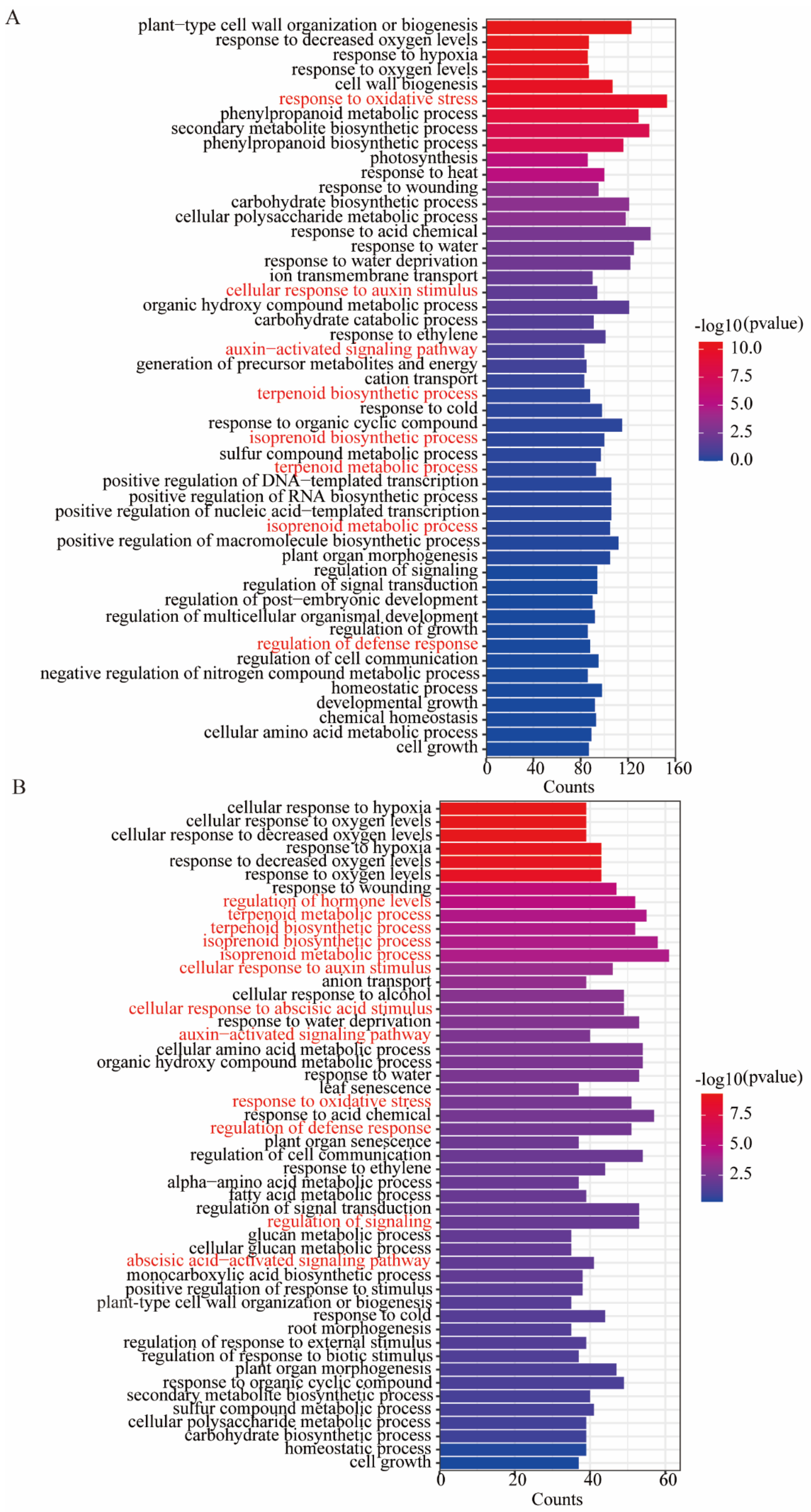

A
plant−type cell wall organization or biogenesis
response to decreased oxygen levels
response to hypoxia
response to oxygen levels
cell wall biogenesis
response to oxidative stress
phenylpropanoid metabolic process
secondary metabolite biosynthetic process
phenylpropanoid biosynthetic process
photosynthesis
response to heat
response to wounding
carbohydrate biosynthetic process
cellular polysaccharide metabolic process
response to acid chemical
response to water
response to water deprivation
ion transmembrane transport
cellular response to auxin stimulus
organic hydroxy compound metabolic process
carbohydrate catabolic process
response to ethylene
auxin−activated signaling pathway
generation of precursor metabolites and energy
cation transport
terpenoid biosynthetic process
response to cold
response to organic cyclic compound
isoprenoid biosynthetic process
sulfur compound metabolic process
terpenoid metabolic process
positive regulation of DNA−templated transcription
positive regulation of RNA biosynthetic process
positive regulation of nucleic acid−templated transcription
isoprenoid metabolic process
positive regulation of macromolecule biosynthetic process
plant organ morphogenesis
regulation of signaling
regulation of signal transduction
regulation of post−embryonic development
regulation of multicellular organismal development
regulation of growth
regulation of defense response
regulation of cell communication
negative regulation of nitrogen compound metabolic process
homeostatic process
developmental growth
chemical homeostasis
cellular amino acid metabolic process
cell growth
0
40
80
120
160
Counts
-log10(pvalue)
10.0
7.5
5.0
2.5
0.0
B
cellular response to hypoxia
cellular response to oxygen levels
cellular response to decreased oxygen levels
response to hypoxia
response to decreased oxygen levels
response to oxygen levels
response to wounding
regulation of hormone levels
terpenoid metabolic process
terpenoid biosynthetic process
isoprenoid biosynthetic process
isoprenoid metabolic process
cellular response to auxin stimulus
anion transport
cellular response to alcohol
cellular response to abscisic acid stimulus
response to water deprivation
auxin−activated signaling pathway
cellular amino acid metabolic process
organic hydroxy compound metabolic process
response to water
leaf senescence
response to oxidative stress
response to acid chemical
regulation of defense response
plant organ senescence
regulation of cell communication
response to ethylene
alpha−amino acid metabolic process
fatty acid metabolic process
regulation of signal transduction
regulation of signaling
glucan metabolic process
cellular glucan metabolic process
abscisic acid−activated signaling pathway
monocarboxylic acid biosynthetic process
positive regulation of response to stimulus
plant−type cell wall organization or biogenesis
response to cold
root morphogenesis
regulation of response to external stimulus
regulation of response to biotic stimulus
plant organ morphogenesis
response to organic cyclic compound
secondary metabolite biosynthetic process
sulfur compound metabolic process
cellular polysaccharide metabolic process
carbohydrate biosynthetic process
homeostatic process
cell growth
0
20
40
60
Counts
-log10(pvalue)
7.5
5.0
2.5

Fig.S3 Top 40 Gene Ontology biological process terms enriched among upregulated differentially expressed genes (DEGs) in hairy roots. (A) HR versus CK and (B) HR+MeJA versus HR. The color gradient represents the enrichment significance as −log10(*p*-value), and the bar length indicates the number of enriched genes.

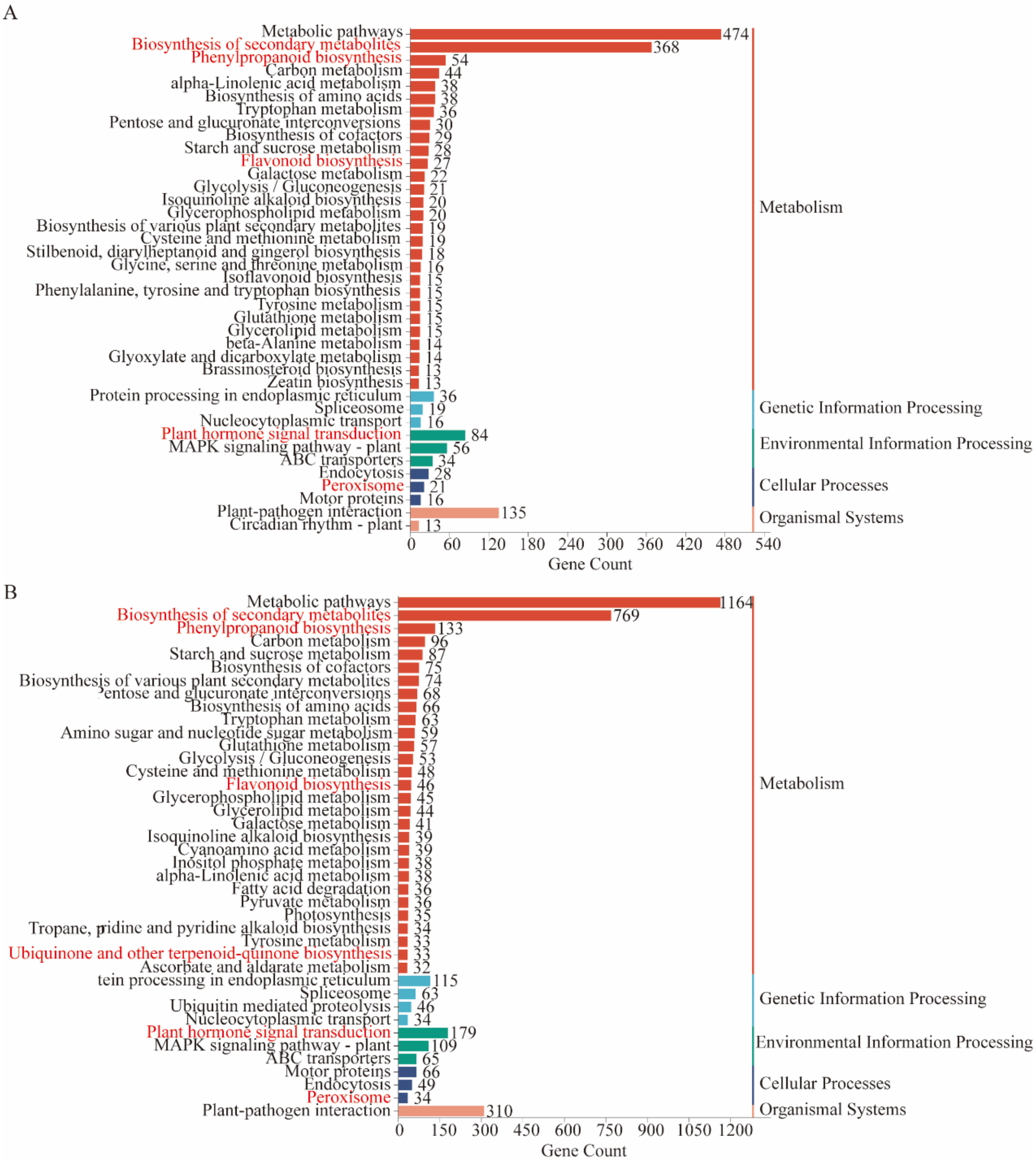

Fig. S4 Top 40 Kyoto Encyclopedia of Genes and Genomes (KEGG) pathways enriched among differentially expressed genes (DEGs) in hairy roots. (A) HR versus CK and (B) HR+MeJA versus HR. The bar length indicates the number of genes assigned to each pathway.

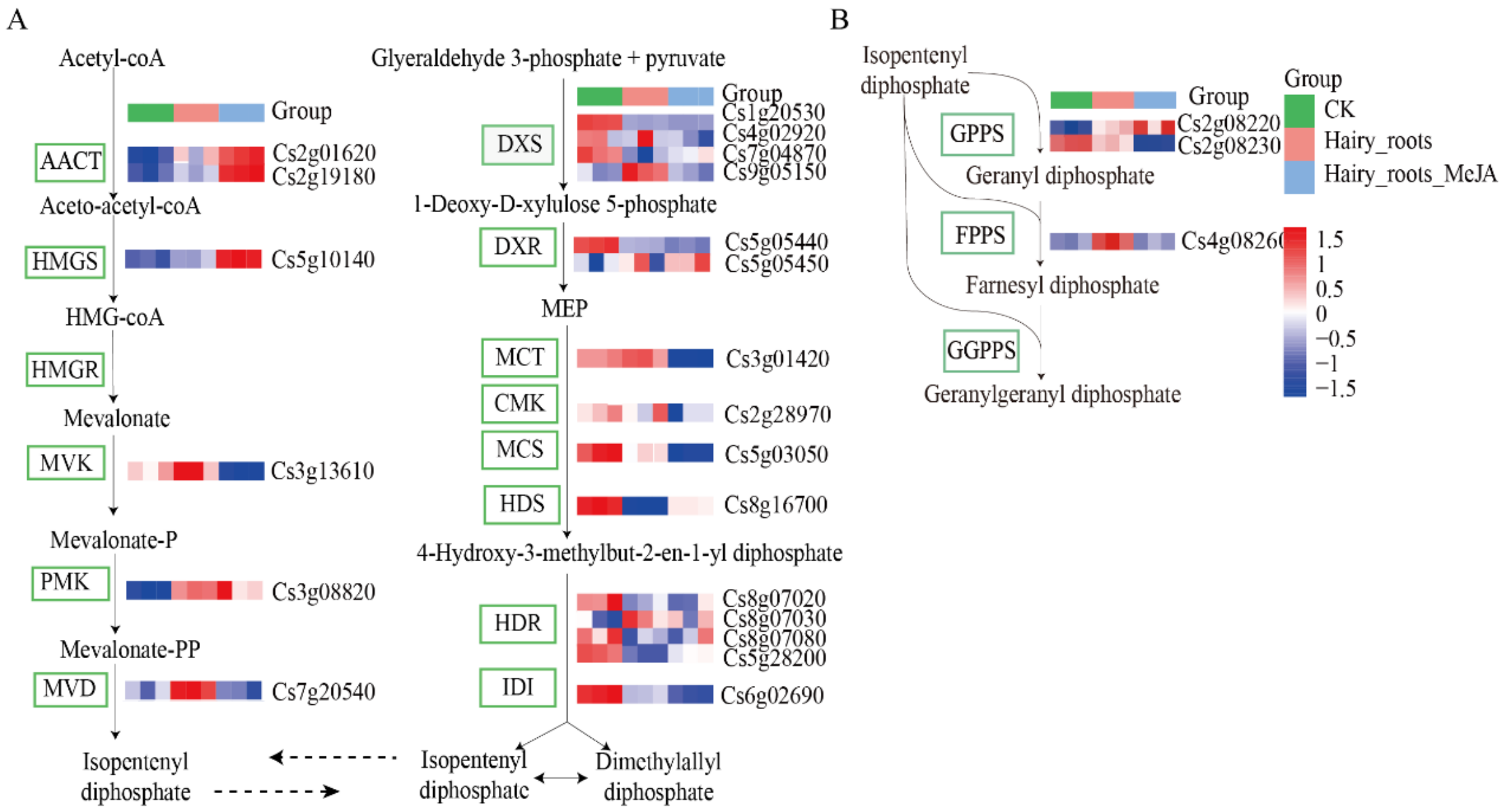

Fig. S5 Heatmap of the differentially expressed terpenoid biosynthetic module genes in citrange hairy roots (HR) induced by *Rhizobium rhizogenes* (*R. rhizogenes*) and that stimulated by methyl jasmonate (MeJA) treatment (HR+MeJA). (A) Genes encoding proteins related to 2-C-methylerythritol-4-phosphate (MEP), mevalonate (MVA) and isoprenoid submodules. (B) Genes encoding proteins related to prenyl transferase and terpene synthase submodules. Values are the log2-fold changes compared with the average expression in noninduced roots. Light red indicates upregulation and dark red indicates strong upregulation of gene expression. Blue indicates downregulation of gene expression levels and a strong downregulation is indicated in dark blue. transcript per million mapped reads (FPKM) values are presented in Supporting Information Table S5. AACT: acetoacetyl-CoA thiolase; HMGS: 3-hydroxy-3-methylglutaryl-CoA synthase; HMGR: HMG-CoA reductase; MVK: mevalonate kinase; PMK: phosphomevalonate kinase; MVD: mevalonate diphosphate decarboxylase; DXS:1-deoxy-d-xylulose 5- phosphate synthase; DXR: 1-deoxy-d-xylulose 5-phosphate reductoisomerase; MCT, 2-C-methyl-D-erythritol 4-phosphate cytidylyltransferase; CMK, 4-(cytidine 5-diphospho)-2-C-methyl-D-erythritol kinase; MCS: 2-C-methyl-D-erythritol 2,4-cyclodiphosphate synthase; HDS, 1-hydroxy-2-methyl- 2-butenyl 4-diphosphate synthase; HDR, 4-hydroxy-3-methylbut-2-enyldiphosphatereductase; IDI: isopentenyl diphosphate isomerase; GPPS, geranyl pyrophosphate synthase; FPPS: farnesyl pyrophosphate synthase; GPPS: geranyl pyrophosphate synthase; GGPPS: geranylgeranyl diphosphate synthase.

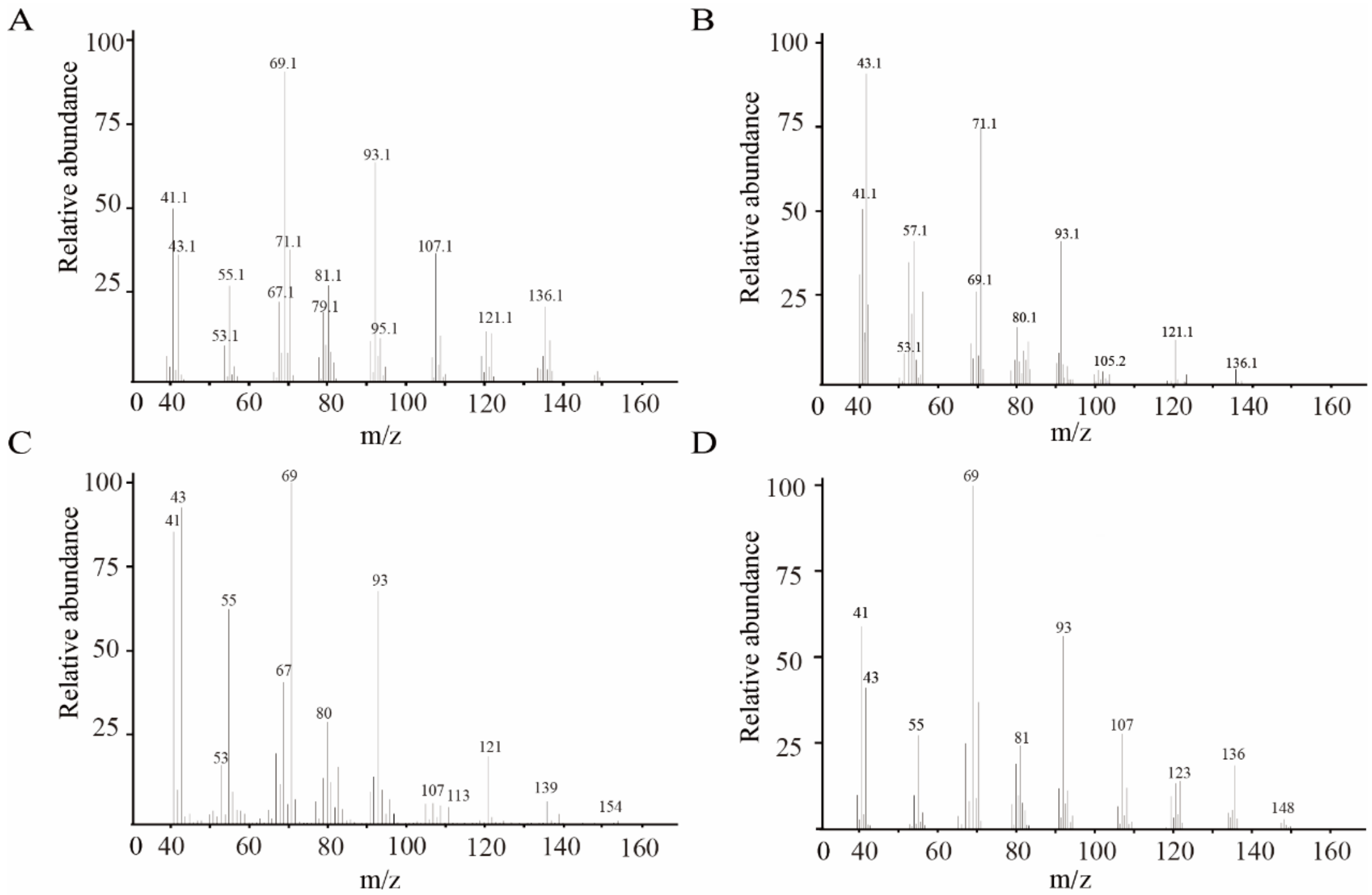

Fig. S6 Mass spectra comparisons of the *in vitro* enzymatic products against authentic chemical standards. (A) Mass spectrum of the product derived from *Cs2g07240*+GPP incubation; (B) Mass spectrum of the product derived from *Cs2g07240*+FPP incubation; (C) Mass spectrum of the authentic linalool standard; (D) Mass spectrum of the authentic trans-nerolidol standard.

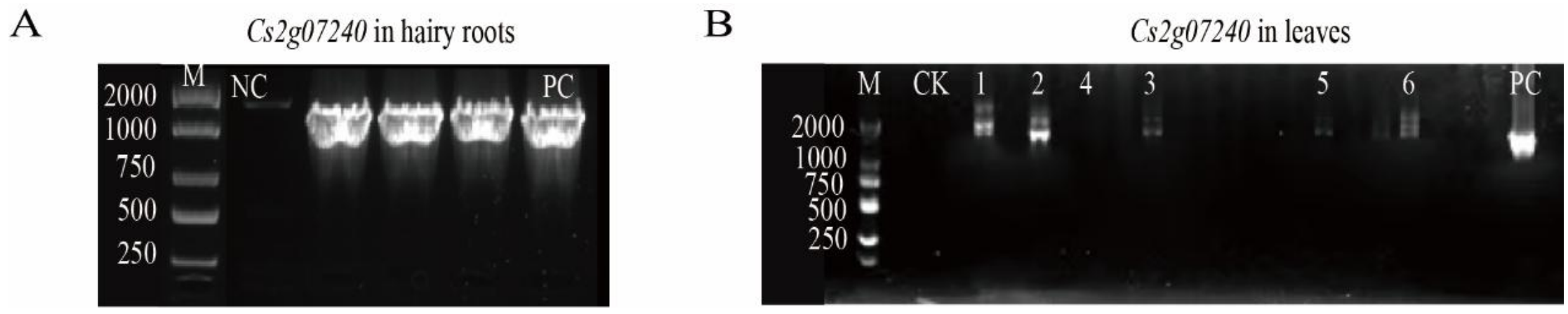

Fig. S7 PCR detection of *Cs2g07240* in transgenic citrus hairy roots and leaves. PCR analyses for the presence of exogenous *Cs2g07240* in the transgenic citrange roots(A) and in in the transgenic citrange leaves. M: DNA mark D2000; PC: plasmid DNA as positive control; NC: non-transformed citrange root DNA as negative control; CK: negative control (non-transgenic leaf DNA); Lanes 1–3: individual transgenic leaf lines overexpressing *Cs2g07240*.
